# Regulation of pairing between broken DNA-containing chromatin regions by Ku80, DNA-PKcs, ATM, and 53BP1

**DOI:** 10.1038/srep41812

**Published:** 2017-02-03

**Authors:** Motohiro Yamauchi, Atsushi Shibata, Keiji Suzuki, Masatoshi Suzuki, Atsuko Niimi, Hisayoshi Kondo, Miwa Miura, Miyako Hirakawa, Keiko Tsujita, Shunichi Yamashita, Naoki Matsuda

**Affiliations:** 1 Department of Radiation Biology and Protection, Atomic Bomb Disease Institute, Nagasaki University, 1-12-4 Sakamoto, Nagasaki, 852-8523, Japan; 2 Advanced Scientific Research Leaders Development Unit, Gunma University, 3-39-22 Showa-machi, Maebashi, Gunma, 371-8511, Japan; 3 Department of Radiation Medical Sciences, Atomic Bomb Disease Institute, Nagasaki University, 1-12-4 Sakamoto, Nagasaki, 852-8523, Japan; 4 Department of Pathology, Institute of Development, Aging and Cancer, Tohoku University, 4-1 Seiryo-machi, Aoba-ku Sendai, Miyagi, 980-8575, Japan; 5 Research Program for Heavy Ion Therapy, Division of Integrated Oncology Research, Gunma University Initiative for Advanced Research (GIAR), 3-39-22 Showa-machi, Maebashi, Gunma, 371-8511, Japan; 6 Department of Global Health, Medicine and Welfare, Atomic Bomb Disease Institute, Nagasaki University, 1-12-4 Sakamoto, Nagasaki, 852-8523, Japan; 7 Radioisotope Research Center, Life Science Support Center, Nagasaki University, 1-12-4 Sakamoto, Nagasaki, 852-8523, Japan; 8 School of Medicine, Nagasaki University, 1-12-4 Sakamoto, Nagasaki, 852-8523, Japan

## Abstract

Chromosome rearrangement is clinically and physiologically important because it can produce oncogenic fusion genes. Chromosome rearrangement requires DNA double-strand breaks (DSBs) at two genomic locations and misrejoining between the DSBs. Before DSB misrejoining, two DSB-containing chromatin regions move and pair with each other; however, the molecular mechanism underlying this process is largely unknown. We performed a spatiotemporal analysis of ionizing radiation-induced foci of p53-binding protein 1 (53BP1), a marker for DSB-containing chromatin. We found that some 53BP1 foci were paired, indicating that the two damaged chromatin regions neighboured one another. We searched for factors regulating the foci pairing and found that the number of paired foci increased when Ku80, DNA-PKcs, or ATM was absent. In contrast, 53BP1 depletion reduced the number of paired foci and dicentric chromosomes—an interchromosomal rearrangement. Foci were paired more frequently in heterochromatin than in euchromatin in control cells. Additionally, the reduced foci pairing in 53BP1-depleted cells was rescued by concomitant depletion of a heterochromatin building factor such as Krüppel-associated box-associated protein 1 or chromodomain helicase DNA-binding protein 3. These findings indicate that pairing between DSB-containing chromatin regions was suppressed by Ku80, DNA-PKcs, and ATM, and this pairing was promoted by 53BP1 through chromatin relaxation.

Chromosome rearrangements (CRs), such as inversions and translocations, are often found in hematopoietic malignancies and solid tumours^1^. CR can lead to the juxtaposition of proto-oncogenes (*e.g., c-myc*) with strong enhancers of other genes (*e.g.,* immunoglobulin genes) or the generation of chimeric fusion genes such as *BCR-ABL1*^2^. As a result, CR gives rise to deregulated expression or constitutive activation of proto-oncogene products. CR can also drive carcinogenesis by interrupting the sequences of tumour suppressor genes, such as that of tetratricopeptide repeat domain 28 in colorectal cancer^3^. Despite the clinical and physiological importance of CRs, the molecular mechanism underlying CR remains to be elucidated.

CR formation is initiated by the concomitant occurrence of DNA double-strand breaks (DSBs) at two or more genomic locations. A recent study showed that DSB-containing chromatin regions move and pair with each other before chromosomal translocation, indicating that movement and pairing of damaged chromatin regions are both necessary processes for CR^4^. Although chromatin is intrinsically mobile, whether DSBs enhance chromatin mobility is a matter of debate^5^,^6^. Several studies using mammalian cells have shown that DSB-containing chromatin exhibits limited mobility with a mean squared displacement (MSD) of ≤1 μm^2^/h, which is comparable to the mobility of undamaged chromatin^4^,^7^,^8^. Another study, however, showed that ionizing radiation (IR)-induced foci of p53-binding protein 1 (53BP1)-green fluorescent protein (GFP), which is a marker of DSB-containing chromatin, were slightly but significantly more mobile than undamaged chromatin domains^9^. Despite the conflicting results described above, there seems to be a consensus that most DSBs in mammalian cells undergo limited motion with an MSD of ≤1 μm^2^/h, irrespective of the methods employed for DSB induction, including the use of γ-rays, heavy ions, or I-Sce I endonuclease^4^,^7^,^9^.

DSB movement can cause pairing between DSB-containing chromatin regions, which is an event that occurs before CR^4^. Factors regulating the pairing process, however, are largely unknown, except for MRE11, which has been shown to promote pairing between chromatin regions containing DSBs that were site-specifically induced by I-Sce I endonuclease^4^. So far, no other factors have been identified that regulate this pairing.

In the present study, we aimed to elucidate how pairing between damaged chromatin regions occurs and what factors regulate this process. We used IR-induced 53BP1 foci as an indicator of DSB-containing chromatin regions and found some foci that were paired. Live cell imaging of IR-induced foci revealed that most of the paired foci formed via dynamic pairing—pairing between separate foci by movement of the foci. By analysing the paired-foci frequency, we identified several factors that suppress or promote foci pairing and obtained mechanistic insight into how chromatin relaxation influences foci pairing.

## Results

### Pairing between ionizing radiation-induced foci

**Figure 1:**
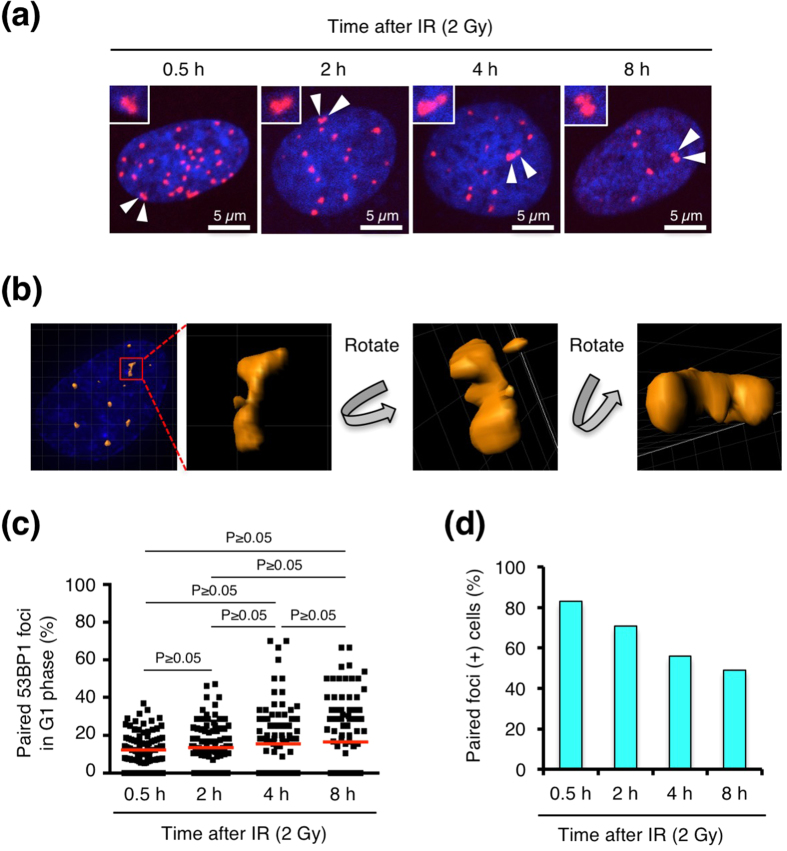
Pairing of IR-induced foci. (**a**) BJ-hTERT cells were irradiated with 2 Gy IR and fixed at the indicated time points. EdU was applied for 30 min before fixation to label S phase cells. After fixation, immunofluorescence staining was performed to visualize 53BP1 and phospho-H3 (a G2/M marker), followed by EdU detection. The images show 53BP1 foci (red) in EdU(−)/phospho-H3(−) cells (G1 phase cells). Nuclei were counterstained with 4′,6-diamidino-2-phenylindole (DAPI, blue). The white arrowheads represent paired foci (enlarged in insets). (**b**) Three-dimensional image of paired foci, which was obtained as described in *Methods*. Rotation of the image confirmed the three-dimensional pairing of foci. (**c**) Frequency of paired 53BP1 foci in BJ-hTERT cells (G1 phase). Plotted are the percentages of paired foci among the total foci in individual cells. Red bars represent means. The statistical comparison was performed using the Dunn’s multiple comparison test (alpha = 0.05). (**d**) Percentage of paired foci (+) cells. Paired foci (+) cells were counted in 100 cells at each time point after IR. IR, ionizing radiation; 53BP1, p53-binding protein 1.

In this study, we used IR to induce DSBs randomly in the genome. Immunofluorescence staining of 53BP1 was performed to visualise DSB-containing chromatin, because it is generally accepted that 53BP1 forms foci at DSB sites^10^. We confirmed that 53BP1 foci colocalised with the foci of serine 139-phosphorylated histone H2AX, a well-established marker of DSB sites (Supplementary Fig. S1)^11^. We observed 53BP1 foci at several time points after exposure to IR in BJ-hTERT cells and found that some of the 53BP1 foci were paired (Fig. 1a). Three-dimensional imaging confirmed foci pairing from 360° (Fig. 1b and Supplementary Movie S1). Based on the results shown in Fig. 1a and b, we decided to use paired 53BP1 foci as an indicator of pairing between damaged chromatin regions in subsequent experiments. We next analysed the frequency of paired foci. Paired foci in G1 phase cells were counted, because our preliminary experiments suggested that IR-induced dicentric chromosomes, a type of CR, mostly originated in the G0/G1 phase (Supplementary Fig. S2a). To identify G1 phase cells, 53BP1 was co-stained with 5-ethynyl-2′-deoxyuridine (EdU, an S phase marker) and serine 10-phosphorylated histone H3 (phospho-H3, a G2/M phase marker), and EdU(−)/phospho-H3(−) cells were subjected to foci-pairing analyses (Supplementary Fig. S2b). Fig. 1c and Supplementary Table S1 show the percentage of paired foci in individual cells and the number of paired foci out of all foci scored in cells, respectively. The frequency of paired foci varied between cells, but on average, 10–20% of the total foci were paired at all time points examined (Fig. 1c). Although the number of cells with a high foci-pairing frequency (>40%) appeared to increase with time after IR, there was no statistically significant difference between time points when total cells including paired foci(−) cells were analysed (Fig. 1c). To determine whether long-remaining foci tend to pair, we next analysed paired-foci frequency only in paired foci(+) cells (Supplementary Fig. S3a). Unlike the result in Fig. 1c, paired-foci frequency increased with time after IR, while foci number decreased similarly in total- and paired foci(+) cells after IR (Supplementary Fig. S3a, b). We next examined the effect of foci number on paired-foci frequency by irradiating with varying doses of IR. Analysis in paired foci(+) cells at 8 h after IR showed that paired-foci frequency decreased with IR dose, while foci number increased with IR dose both in total and paired foci(+) cells similarly (Supplementary Fig. S3c,d). When total cells including paired foci(−) cells were analysed, the paired-foci frequency did not differ significantly between IR doses, although cells with a higher foci-pairing frequency apparently decreased with dose (Supplementary Fig. S3e). Based on the results in Fig. 1c and Supplementary Fig. S3a–e, we conclude that long-remaining foci do not have a notable tendency to pair, and that the apparent increase in cells with a higher paired-foci frequency at later time points after IR may be attributable to a DSB-repair-dependent decrease in foci number.

**Figure 2:**
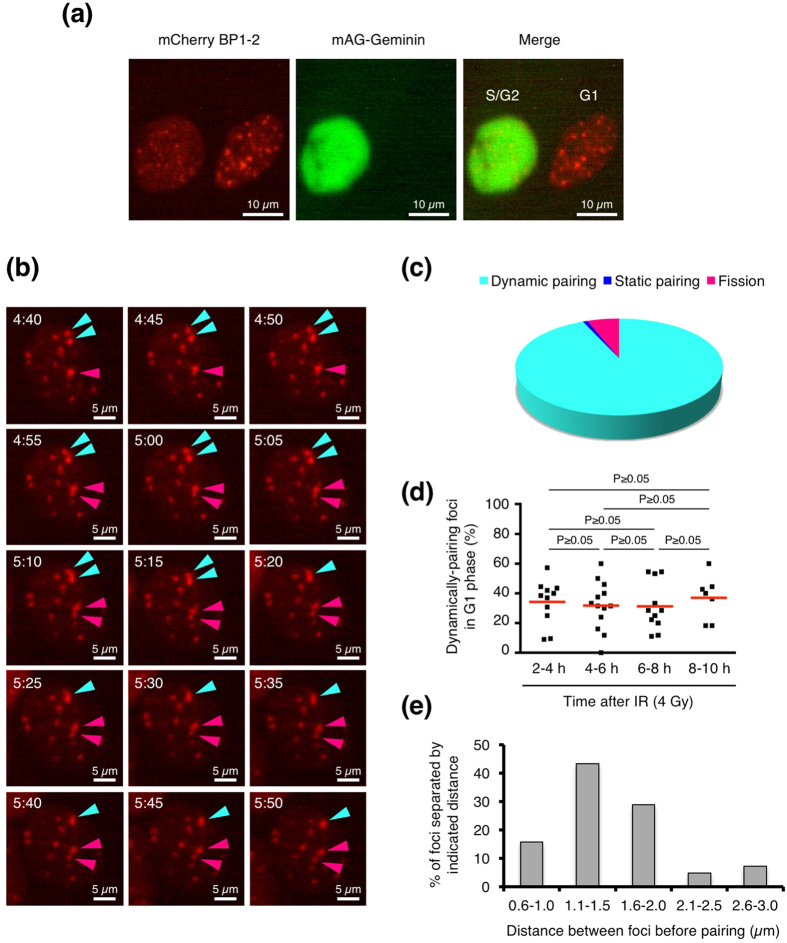
Foci pairing in live cells. (**a**) Typical images of mCherry-BP1-2 foci and mAG-Geminin in BJ-hTERT cells exposed to IR. Since mAG-Geminin is a S/G2/M phase marker, the mAG-Geminin(+) cell (left) is indicated as S/G2. The mAG-Geminin(−) cell (right) is indicated as G1. (**b**) Series of images showing mCherry-BP1-2 foci in a living BJ-hTERT cell (G1 phase). Light blue arrowheads indicate foci that underwent “dynamic pairing”, and pink arrowheads indicate foci that underwent “fission”. The numbers at the top left indicate the time after IR (hr:min). (**c**) Pie chart showing the percentage of mCherry-BP1-2 foci that exhibited each foci pairing pattern (dynamic pairing, static pairing, or fission). (**d**) Frequency of mCherry-BP1-2 foci that underwent dynamic pairing in living BJ-hTERT cells (G1 phase). Plotted are the percentages of dynamically pairing foci among the total foci in individual cells during each 2-h time interval (*e.g*., 2–4 h). Red bars represent means. The statistical comparison was performed using the Dunn’s multiple comparison test (alpha = 0.05). (**e**) The distances between foci before dynamic pairing. The distances were measured using the captured live cell images. IR, ionizing radiation.

We next examined how foci pairing occurs in live cells. To observe foci pairing in live cells, we utilized mCherry-BP1-2, a fusion protein of mCherry and the minimal focus-forming region of 53BP1^12^. Previous studies have confirmed that this mCherry fusion protein can accumulate at DSB sites, but it is neither functional nor does it disturb endogenous 53BP1^12^,^13^. We also used mAG-Geminin as an S/G2/M phase marker to identify live cells in the G1 phase^14^. We generated BJ-hTERT cells that stably express both mCherry-BP1-2 and mAG-Geminin (Fig. 2a). We confirmed that the behaviour of mCherry-BP1-2 foci was identical to that of endogenous 53BP1 foci in terms of the foci kinetics and percentage of paired foci in the G1 phase after 2 Gy IR (foci number: Supplementary Fig. S3f and S3g; foci pairing: Fig. 1c and Supplementary Fig. S3h). We performed live cell imaging of mCherry-BP1-2 foci and found that foci pairing occurred by the following three means: dynamic pairing (pairing between separate foci via foci movement; Fig. 2b, Supplementary Fig. S4a,b), static pairing (continuous foci pairing; Supplementary Fig. S4c), and fission (division of a single focus into two foci; Fig. 2b). Analysis of live cell images revealed that >93% of foci-pairing events occurred via dynamic pairing (Fig. 2c). We next determined the frequency of dynamic pairing (Fig. 2d). Foci pairing was examined in 2-h time frames from 2 h to 10 h after exposure to IR (*e.g*., 2–4 h), because very few cells remained within a field with time frames longer than 2 h. The frequency of dynamically pairing foci in each cell varied between 0% and 60%, but on average, 35–40% of the total foci underwent dynamic pairing during each time frame (Fig. 2d). The frequency of dynamic foci pairing in live cells did not differ between time frames. Moreover, we measured the distances between foci before dynamic pairing. We found that >85% of the paired foci were located within 2.0 μm of each other before pairing, but foci that were as far apart as 3.0 μm also moved and paired (Fig. 2e).

### Factors that regulate foci pairing

**Figure 3:**
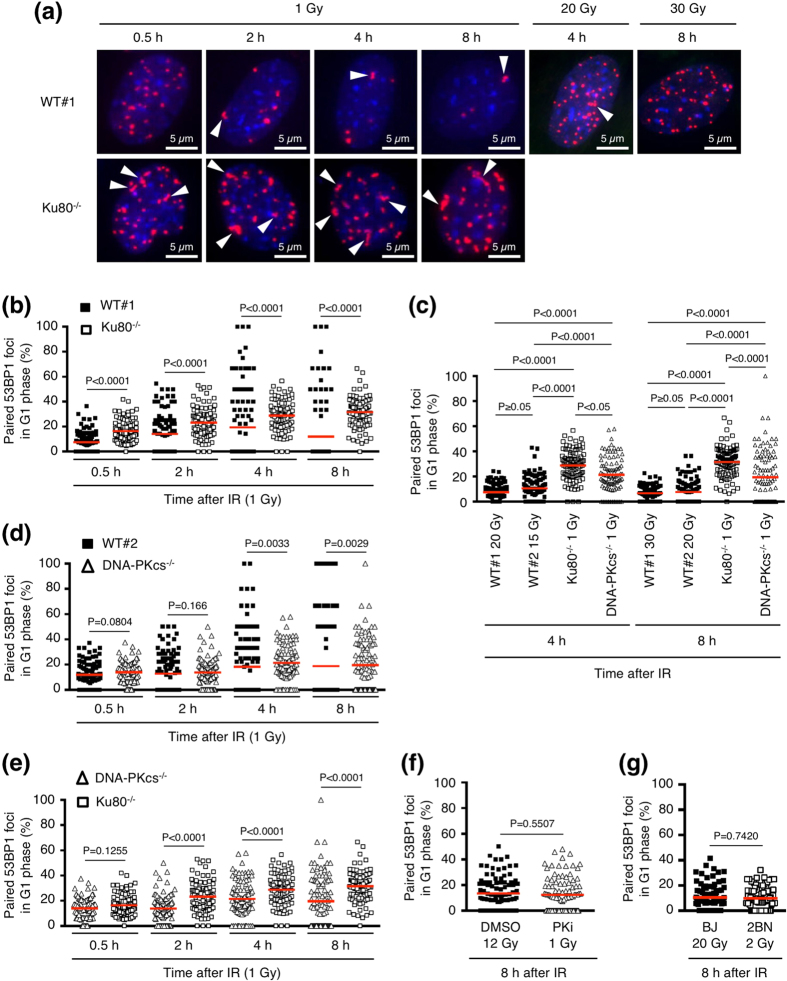
Effects of classical non-homologous end-joining factors on foci pairing. (**a**) Images of IR-induced 53BP1 foci (red) in WT#1 and Ku80^−/−^ MEFs (G1 phase). Cells were irradiated with the indicated doses of IR and fixed at the indicated times after IR. Nuclei were counterstained with 4′,6-diamidino-2-phenylindole (DAPI, blue). White arrowheads indicate the pairing of two or more foci. (**b**) Frequency of paired 53BP1 foci in WT#1 and Ku80^−/−^ MEFs (G1 phase) after 1 Gy IR. (**c**) Comparison of the paired-foci frequency between 1 Gy-irradiated Ku80^−/−^ MEFs, 1 Gy-irradiated DNA-PKcs^−/−^ MEFs, and two WT MEFs (WT#1 and #2) irradiated with 15–30 Gy. (**d**) Frequency of paired 53BP1 foci in WT#2 and DNA-PKcs^−/−^ MEFs (G1 phase) after 1 Gy IR. (**e**) Comparison of the paired-foci frequency in Ku80^−/−^ MEFs and DNA-PKcs^−/−^ MEFs. The data were extracted from Fig. 3b (Ku80^−/−^ MEFs) and Fig. 3d (DNA-PKcs^−/−^ MEFs). (**f**) Effect of chemical inhibition of DNA-PKcs on 53BP1 foci pairing. BJ-hTERT cells were irradiated with the indicated doses of IR and fixed 8 h later. DNA-PK inhibitor (NU7441, 10 μM) or vehicle (dimethyl sulphoxide) was applied 30 min before IR exposure until the time of fixation. (**g**) Frequency of paired 53BP1 foci in 2 Gy-irradiated 2BN-hTERT cells (XLF-deficient) and 20 Gy-irradiated BJ-hTERT cells. Plotted in Fig. 3b–g are the percentages of paired foci among the total foci in individual cells. Red bars in Fig. 3b–g represent means. The statistical comparisons shown in Fig. 3b,d–g were performed using the two-tailed Mann-Whitney test (alpha = 0.05). The statistical comparisons shown in Fig. 3c was performed using the Dunn’s multiple comparison test (alpha = 0.05). IR, ionizing radiation; 53BP1, p53-binding protein 1; MEF, mouse embryonic fibroblast; WT, wild-type.

Next, we searched for factors that regulate foci pairing. We first examined factors involved in classical non-homologous end-joining (C-NHEJ)—a major DSB repair pathway—because several studies have shown that defects in the C-NHEJ pathway increase the frequency of CR^15^,^16^,^17^. C-NHEJ factors include Ku70, Ku80, the catalytic subunit of DNA-PK (DNA-PKcs), XLF, XRCC4, and ligase IV^18^. We first examined the role of Ku80 in foci pairing using Ku80^−/−^ mouse embryonic fibroblasts (MEFs). In Ku80^−/−^ MEFs, the 53BP1 foci disappeared much more slowly than those in the wild-type MEFs (WT#1), reflecting the severe defect in DSB repair in the absence of Ku80 (Fig. 3a and Supplementary Fig. S5a). We frequently observed paired foci in Ku80^−/−^ cells (Fig. 3a). Fig. 3b and Supplementary Table S2 show the percentage of foci that were paired in individual cells and the sum of the paired or total foci in all cells examined, respectively. After 1 Gy IR, the frequency of paired foci in Ku80^−/−^ cells was significantly higher than that in WT#1 cells at all time points examined (Fig. 3b). Some WT#1 cells at 4 h or 8 h after 1 Gy IR showed a high frequency (>50%) of paired foci; however, this is likely attributable to small numbers of total foci at these time points in WT#1 cells (see foci number kinetics in WT#1 and Ku80^−/−^ cells in Supplementary Fig. S5a). Paired foci(+) cells among WT cells were much less than those among Ku80^−/−^ cells (Supplementary Table S2). To examine whether the increased foci pairing in Ku80^−/−^ cells was caused by many residual foci in these cells, we next compared foci pairing in 1 Gy-irradiated Ku80^–/–^ cells and WT cells irradiated with high doses of IR. Compared to the foci number in 1 Gy-irradiated Ku80^−/−^ cells, greater or comparable number of residual foci was observed in WT#1 cells or WT#2 cells at 4 h or 8 h after exposure to 15–30 Gy of IR (Supplementary Fig. S5a). The paired-foci frequency in 1 Gy-irradiated Ku80^−/−^ cells was higher than that in WT#1 or WT#2 cells irradiated with the high doses of IR (Fig. 3c). We next examined the role of DNA-PKcs in foci pairing using DNA-PKcs^−/−^ MEFs. The DSB repair defect in DNA-PKcs^−/−^ MEFs was milder than that in Ku80^−/−^ MEFs (Supplementary Fig. S5a). The percentage of paired foci in individual cells and the sum of the paired or total foci in all scored cells are shown in Fig. 3d and Supplementary Table S3, respectively. The paired-foci frequency did not differ between WT#2 and DNA-PKcs^−/−^ cells at 0.5 h and 2 h after 1-Gy IR, but more paired foci were observed in DNA-PKcs^−/−^ cells at 4 h and 8 h compared with WT#2 cells at the same time points (Fig. 3d). The frequency of paired foci in 1 Gy-irradiated DNA-PKcs^−/−^ cells was also higher than that in WT#1 or #2 cells irradiated with 15–30 Gy of IR (Fig. 3c). However, the paired-foci frequency in DNA-PKcs^−/−^ cells was lower than that in Ku80^−/−^ cells at 2–8 h after IR (Fig. 3c,e). We also examined the role of DNA-PKcs kinase activity using an established DNA-PKcs inhibitor, NU7441^19^. Paired-foci frequency was not affected by the treatment with 10 μM NU7441—the condition which caused much more residual foci of serine 139-phosphorylated histone H2AX in G1 phase at 24 h after IR, compared with that of the control (Fig. 3f and Supplementary Fig. S5b). Next, we investigated the role of XLF, which interacts with XRCC4/ligase IV and facilitates C-NHEJ^20^. In XLF-deficient 2BN-hTERT cells, the 53BP1 foci decreased much more slowly than those in the control BJ-hTERT cells, reflecting the severely compromised DSB repair in these cells (Supplementary Fig. S5c). However, unlike cells lacking Ku80 or DNA-PKcs, we did not observe increased foci pairing in 2BN-hTERT cells (Fig. 3g, Supplementary Fig. S5d, and Supplementary Table S4).

**Figure 4:**
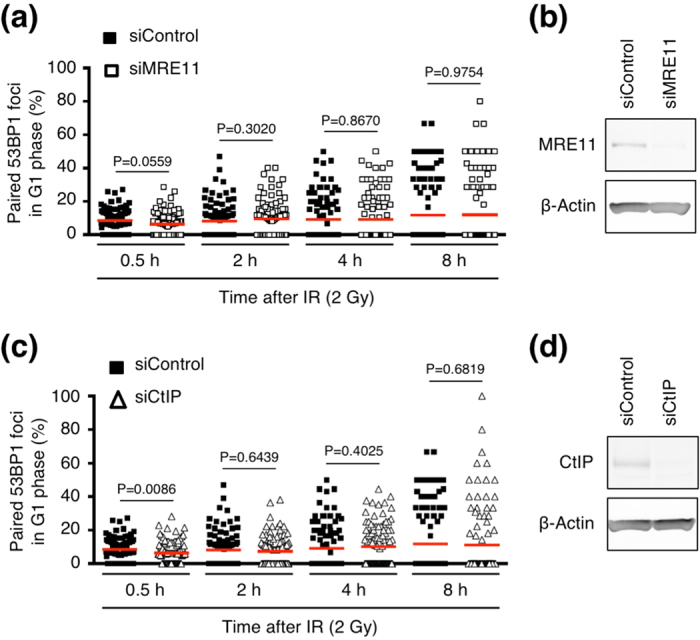
Effects of resection factors on foci pairing. (**a**) Frequency of paired 53BP1 foci in MRE11-depleted cells (G1 phase). BJ-hTERT cells were transfected with the indicated siRNA, and after 3 days the cells were irradiated with 2 Gy ionizing radiation and then fixed at the indicated time points. Plotted are the percentages of paired foci among the total foci in individual cells. Statistical analyses were performed using the two-tailed Mann-Whitney test (alpha = 0.05). Red bars represent means. (**b**) Confirmation of MRE11 depletion by western blotting (cropped blot). (**c**) Frequency of paired 53BP1 foci in CtIP-depleted cells (G1 phase). Transfection of siRNA, sample preparation, examination of foci pairing, and statistical analyses were performed as described for the MRE11-depleted cells in Fig. 4a. (**d**) Confirmation of CtIP depletion by western blotting (cropped blot). Full-length blots for Fig. 4b and d are shown in Supplementary Figure S7a,b and S7c,d, respectively.

We next examined the effect of factors involved in DSB end resection because several studies have shown that alternative NHEJ (A-NHEJ)—a resection-dependent DSB repair pathway—is involved in CR^21^,^22^. However, the depletion of resection factors such as MRE11 and CtIP did not noticeably affect the paired-foci frequency, indicating that resection does not influence pairing between DSB-containing chromatin regions (Fig. 4a–d and Supplementary Table S5).

**Figure 5:**
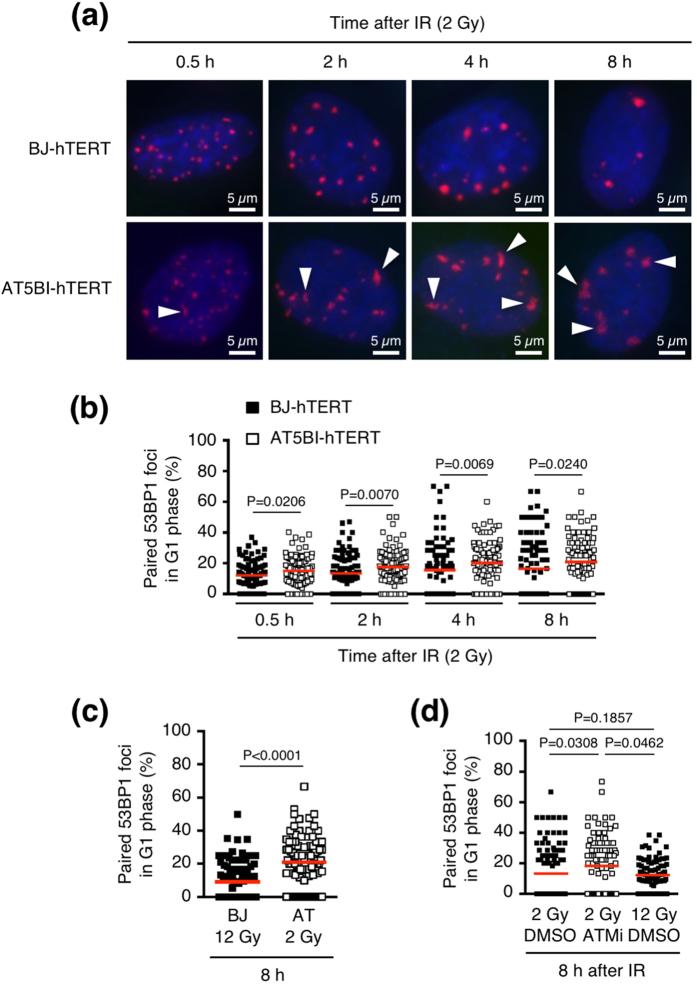
Effects of ATM on foci pairing. (**a**) Images of IR-induced 53BP1 foci (red) in BJ-hTERT and AT5BI-hTERT (ATM-deficient) cells (G1 phase). Cells were irradiated with 2 Gy IR and fixed at the indicated times after IR exposure. Nuclei were counterstained with 4′,6-diamidino-2-phenylindole (DAPI, blue). White arrowheads indicate paired foci. (**b**) Frequency of paired 53BP1 foci in BJ-hTERT and ATM5BI-hTERT cells (G1 phase) after 2 Gy IR. (**c**) Frequency of paired 53BP1 foci in 2 Gy-irradiated AT5BI-hTERT and 12 Gy-irradiated BJ-hTERT cells. (**d**) Effect of chemical inhibition of ATM on foci pairing. Confluent 1BR-hTERT cells (normal human fibroblasts) were irradiated with the indicated doses of IR and fixed 8 h later. ATM inhibitor (10 μM) or vehicle (dimethyl sulphoxide) was applied 30 min before exposure to IR until the time of fixation. Confluent cells were used for the experiment; otherwise, ATM inhibition would have allowed G1-irradiated cells to progress to the next cell cycle phase and few G1 cells would have remained at 8 h after IR exposure. Plotted in Fig. 5b–d are the percentages of paired foci among the total foci in individual cells. Red bars in Fig. 5b–d represent means. The statistical comparisons shown in Fig. 5b–d were performed using the two-tailed Mann-Whitney test (alpha = 0.05). ATM, ataxia telangiectasia mutated; IR, ionizing radiation; 53BP1, p53-binding protein 1.

Next, we investigated the role of ATM in foci pairing because previous studies have shown that ATM suppresses translocation frequency^23^,^24^. We compared the frequency of paired 53BP1 foci between BJ-hTERT and AT5BI-hTERT cells (ATM-deficient human fibroblasts). At earlier time points (0.5 h and 2 h), the size and fluorescence intensity of 53BP1 foci in AT5BI-hTERT cells were less than those in BJ-hTERT cells, but they were comparable in these two cell lines at later time points (4 h and 8 h) (Fig. 5a). Figure 5b and Supplementary Table S6 show the percentage of paired foci in individual cells and the sum of the paired or total foci in all scored cells, respectively. We found that the paired-foci frequency was significantly higher in AT5BI-hTERT cells than in BJ-hTERT cells at all time points after 2-Gy IR (Fig. 5b, Supplementary Tables S1 and S6). Because more 53BP1 foci remained in AT5BI-hTERT cells than in BJ-hTERT cells at later time points, we compared the paired-foci frequency between 2 Gy-irradiated AT5BI-hTERT cells and 12 Gy-irradiated BJ-hTERT cells (Fig. 5c and Supplementary Fig. S5e). The paired-foci frequency was higher in the ATM-deficient cells than in the control cells (Fig. 5c). The chemical inhibition of ATM kinase activity also increased paired foci in confluent 1BR-hTERT cells (normal human fibroblasts) (Fig. 5d).

### Involvement of 53BP1 and chromatin relaxation in foci pairing

**Figure 6:**
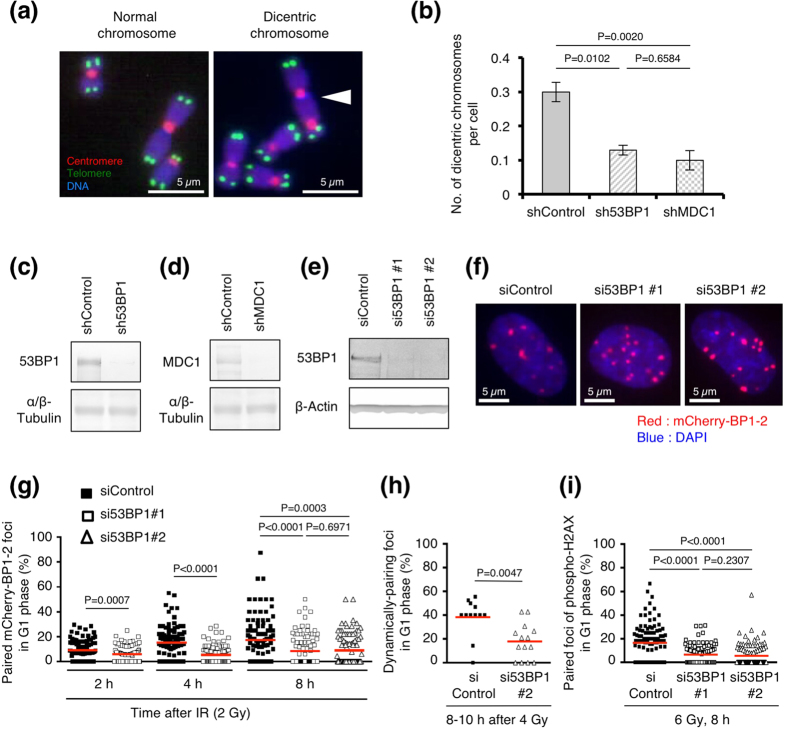
Roles of 53BP1 in chromosome rearrangement and foci pairing. (**a**) Fluorescence *in situ* hybridization of centromeres (red) and telomeres (green). The white arrowhead indicates a dicentric chromosome. Chromosomes were counterstained with DAPI (blue). (**b**) Frequency of IR-induced dicentric chromosomes in BJ-hTERT cells depleted of 53BP1 or MDC1. Statistical analyses were performed using Fisher’s exact test (alpha = 0.05). Results represent the mean ± SD based on two independent experiments. (**c**) Confirmation of shRNA-mediated 53BP1 depletion (Fig. 6b) by western blotting (cropped blot). (**d**) Confirmation of shRNA-mediated MDC1 depletion (Fig. 6b) by western blotting (cropped blot). (**e**) Confirmation of siRNA-mediated 53BP1 depletion in BJ-hTERT cells (Fig. 6f and g) by western blotting (cropped blot). (**f**) Foci of mCherry-BP1-2 in cells transfected with 53BP1 siRNAs. mCherry-BP1-2 foci in BJ-hTERT cells (G1 phase) at 8 h after 6 Gy IR are shown. (**g**) Paired-foci frequency in 53BP1-depleted cells (G1 phase). BJ-hTERT cells that expressed mCherry-BP1-2 and mAG-Geminin were used. Cells were transfected with the indicated siRNA, and after 3 days the cells were irradiated with 2 Gy IR and fixed at the indicated time points. (**h**) Effect of 53BP1 depletion on foci pairing in living cells. BJ-hTERT cells that expressed mCherry-BP1-2BP1-2 and mAG-Geminin were used. Cells were transfected with the indicated siRNA, and after 3 days they were irradiated with 4 Gy IR and subjected to live cell imaging. Foci that exhibited dynamic pairing were scored. (**i**) Frequency of paired phospho-H2AX foci in 53BP1-depleted cells. 1BR-hTERT cells were transfected with the indicated siRNA, and after 3 days the cells were irradiated with 6 Gy IR and fixed 8 h after IR exposure. Plotted in Fig. 6g–i are the percentages of paired foci among the total foci in individual cells. Red bars in Fig. 6g–i represent means. The statistical comparisons shown in Fig. 6g–i were performed using the two-tailed Mann-Whitney test (alpha = 0.05). shRNA, short-hairpin RNA; siRNA, small-interfering RNA. Full-length blots for Fig. 6c–e are shown in Supplementary Figure S8 and S9, respectively.

We next examined the impact of 53BP1 on foci pairing and CR because previous studies have shown that 53BP1 facilitates joining between distal DSB ends during V(D)J or class switch recombination, as well as between unprotected telomeres^12^,^25^,^26^,^27^. We examined the effect of 53BP1 depletion on CR by counting dicentric chromosomes. Analysis by centromere/telomere fluorescence *in situ* hybridization revealed that 53BP1 depletion reduced the incidence of IR-induced dicentric chromosomes (Fig. 6a–c). The depletion of MDC1—a factor required for 53BP1 recruitment to DSB-containing chromatin—also decreased the frequency of IR-induced dicentric chromosomes (Fig. 6b,d). In contrast to dicentric chromosomes, chromosome breaks, which indicate unrepaired DSBs, increased in cells depleted of 53BP1 or MDC1, supporting the notion that 53BP1 and MDC1 are involved in repair of a subset of DSBs (Supplementary Fig. S6a–c)^28^.

Next, we examined the role of 53BP1 in foci pairing. To this end, we utilized mCherry-BP1-2 and 53BP1 siRNAs that do not interfere with the expression of mCherry-BP1-2 (Fig. 6e,f). We confirmed that mCherry-BP1-2 protein formed foci normally in 53BP1 siRNA-treated cells and that the kinetics of mCherry-BP1-2 foci number are similar between 53BP1 siRNA-treated cells and control siRNA-treated cells (Fig. 6f, Supplementary Fig. S6d and e). The percentage of paired foci in individual cells and the sum of the paired or total foci in all scored cells are shown in Fig. 6g and Supplementary Table S7, respectively. We found that 53BP1 depletion significantly reduced the paired-foci frequency at all time points after IR (Fig. 6g and Supplementary Table S7). A reduction in foci pairing by 53BP1 depletion was also observed in living BJ-hTERT cells (Fig. 6h). The pairing of foci of serine 139-phosphorylated histone H2AX was also reduced by 53BP1 depletion (Fig. 6i).

**Figure 7:**
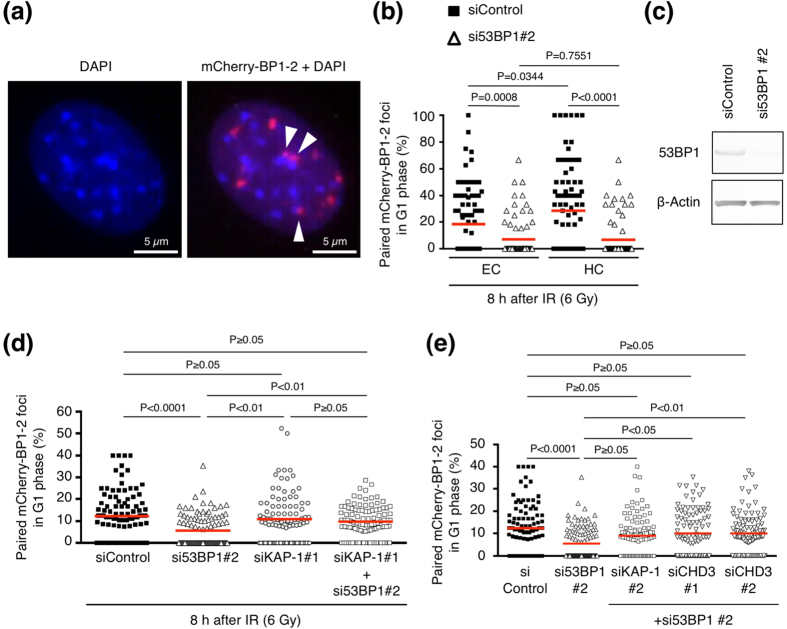
Relationship between chromatin relaxation and 53BP1-dependent foci pairing. (**a**) Typical image of DAPI staining and mCherry-BP1-2 foci in NIH3T3 cells. The nuclear regions with intense DAPI staining correspond to HC. White arrowheads represent “HC foci” that overlap with HC or that are located at the periphery of HC. (**b**) Frequency of paired foci in EC and HC. NIH3T3 cells expressing mCherry-BP1-2BP1-2 were used. Cells were transfected with the indicated siRNA, and after 3 days they were irradiated with 6 Gy IR and then fixed 8 h later. The HC foci were defined as foci overlapping with or at the periphery of HC as shown in Fig. 7a. The EC foci were defined as foci separate from HC. Plotted are the percentages of paired EC foci among the total EC foci or paired HC foci among the total HC foci in individual cells. Note that the frequency of paired foci was significantly higher in HC than in EC in control cells. (**c**) Confirmation of siRNA-mediated 53BP1 depletion in NIH3T3 cells by western blotting (cropped blot). Full-length blots are shown in Supplementary Figure S10. (**d**) Recovery of reduced foci pairing in 53BP1-depleted cells by concomitant depletion of KAP-1, an HC building factor. BJ-hTERT cells that expressed mCherry-BP1-2 and mAG-Geminin were used. Cells were transfected with the indicated siRNA(s), and after 3 days they were irradiated with 6 Gy IR and fixed after 8 h. (**e**) Recovery of reduced foci pairing in 53BP1-depleted cells by concomitant depletion of the HC building factors KAP-1 or CHD3. The samples were prepared as described in the legend of Fig. 7d. Plotted in Fig. 7d and e are the percentages of paired foci among the total foci in individual cells. Red bars in Fig. 7b,d and e represent means. The statistical comparisons in Fig. 7b and in Fig. 7d,e were performed using the two-tailed Mann-Whitney test (alpha = 0.05) and the Dunn’s multiple comparison test (alpha = 0.05), respectively. DAPI, 4′,6-diamidino-2-phenylindole; EC, euchromatin; HC, heterochromatin; IR, ionizing radiation; 53BP1, p53-binding protein 1; KAP-1, Krüppel-associated box-associated protein 1; CHD3, chromodomain helicase DNA-binding protein 3.

A previous study suggested that 53BP1 facilitates the repair of DSBs in heterochromatin (HC) by relaxing DSB-containing HC^28^. Another study showed that 53BP1 promoted the joining of unprotected telomeres, which mimic one-ended DSBs, by increasing chromatin mobility^12^. Because the regions around telomeres form HC, we hypothesized that the movement and pairing of DSB-containing chromatin regions may be promoted with the relaxation of HC. To test this, we examined foci pairing in euchromatin (EC) and HC separately using murine NIH3T3 cells, with HC easily discernible by intense DAPI staining (Fig. 7a)^29^. We generated NIH3T3 cells that stably expressed mCherry-BP1-2 and examined pairing of mCherry-BP1-2 foci in these cells. We defined foci overlapping with or at the periphery of HC as “HC foci” (Fig. 7a). In contrast, foci located separately from HC were defined as “EC foci”. We compared the paired-foci frequency between EC and HC with or without 53BP1 siRNA. We found that in the control cells, the paired-foci frequency in HC was significantly higher than that in EC (Fig. 7b and Supplementary Table S8). The paired-foci frequency decreased in both EC and HC when 53BP1 was depleted (Fig. 7b,c and Supplementary Table S8).

To obtain deeper insight into the relationship between 53BP1-dependent foci pairing and chromatin relaxation, we tested whether the reduced foci pairing in 53BP1-depleted cells could be rescued by inducing chromatin relaxation. To induce chromatin relaxation, we depleted Krüppel-associated box-associated protein 1 (KAP-1)—an HC building factor—because depletion of this protein induces global chromatin relaxation^30^. Although 53BP1 depletion alone reduced the paired-foci frequency, additionally depleting KAP-1 rescued the reduced foci pairing in 53BP1-depleted cells (Fig. 7d and Supplementary Fig. S11,12; the sum of the paired or total foci in all scored cells is shown in Supplementary Table S9). The reduced foci pairing in 53BP1-depleted cells was also rescued by additional treatment of another KAP-1 siRNA (siKAP-1 #2) (Fig. 7e and Supplementary Table S9). To confirm our findings, we explored whether depletion of another HC building factor also rescues reduced foci pairing in 53BP1-depleted cells. As another HC building factor, we chose chromodomain helicase DNA-binding protein 3 (CHD3), whose depletion also relaxes chromatin^30^. We found that the reduced paired-foci frequency in 53BP1-depleted cells was recovered to the level in control cells by additionally depleting CHD3 (Fig. 7e and Supplementary Table S9). In summary, 53BP1 depletion reduced the frequency of dicentric chromosomes and paired foci, and the reduced foci pairing was rescued by concomitant depletion of HC building factors such as KAP-1 and CHD3.

## Discussion

In the present study, we investigated pairings between DSB-containing chromatin regions—the least understood step in CR. We employed paired foci of 53BP1 and mCherry-BP1-2 as indicators of pairing between damaged chromatin regions. Live cell analyses of mCherry-BP1-2 foci revealed that the vast majority of foci pairing events occurred by dynamic pairing—pairing by movement of two or more separate foci. We identified Ku80, DNA-PKcs, and ATM as factors that suppressed foci pairing. We also found that 53BP1 promoted CR and foci pairing, and obtained mechanistic insight into 53BP1-dependent foci pairing.

Our live cell analyses showed that >93% of foci-pairing events were dynamic pairings, not static pairings or fission events. This indicates that most of the paired foci scored in this study occurred via dynamic pairing between two or more separate foci, not by generation of multiple DSBs in close proximity. Previous studies have shown that in mammals, movement of DSB-containing chromatin is limited, with the MSD of damaged chromatin ≤1 μm^2^/h^4^,^7^,^9^. In our study, >85% of dynamic pairings occurred between foci located ≤ 2.0 μm from each other, while pairings between more distal foci were also observed. Given that the MSD of DSB-containing chromatin is ~1 μm^2^/h, it is reasonable that dynamic pairing preferentially occurs between foci that were located within 2.0 μm of each other during the 2-h time frame of our live cell imaging.

In our results, cells with high paired-foci frequency appeared to increase with time after IR. Indeed, paired-foci frequency increased with time after IR when only paired foci(+) cells were analysed (Supplementary Fig. S3a). However, we believe that the apparent time-dependent increase in paired-foci frequency is attributable to a time-dependent decrease in foci number. Namely, if paired-foci numbers were the same between earlier and later time points, the percentage of paired foci would be higher at later time points than at earlier time points because total foci number decreases time-dependently after IR. This notion is supported by the result presented in Supplementary Fig. S3c where the paired-foci frequency at 8 h post-IR was analysed in paired foci(+) cells. This analysis revealed that the paired-foci frequency decreased with increasing IR dose, while foci number increased (Supplementary Fig. S3c and d). If long-remaining foci tended to pair, the paired-foci frequency should not be affected by IR dose or foci number. Paired-foci frequency did not differ between IR doses when total cells including paired foci(−) cells were analysed (Supplementary Fig. S3e). Taken together, we conclude that the apparent time-dependent increase in cells with high paired-foci frequency is due to smaller foci numbers at later time points, rather than the tendency of long-remaining foci to pair.

The important question is how a DSB end finds another DSB end. DNA sequences around IR-induced DSBs are, in most cases, different between heterologous DSBs. Thus, it is unlikely that CR formation depends on long stretches of sequence homology. This notion is supported by the previous studies showing that translocation formation is not mediated by homologous recombination, and it is also consistent with the reports that translocation junctions in human cancer cells show short or no sequence homology^31^,^32^,^33^,^34^,^35^. Accumulating evidence suggests that translocation formation is mainly mediated by non-homologous end-joining (NHEJ) which relies on little or no sequence homology^31^,^36^. Therefore, we think that a DSB end comes in close proximity to another DSB end via chromatin movement and that the two neighbouring DSB ends are rejoined by NHEJ to form CR. We speculate that 53BP1 promotes movement of DSB-containing chromatin and thereby increases pairing and subsequent NHEJ-dependent rejoining between heterologous DSBs based on the following reasons: (1) mobility of IR-induced mCherry-BP1-2 foci was impaired in 53BP1^−/−^ cells^13^; (2) pairing of mCherry-BP1-2 foci was decreased in 53BP1-depleted cells (our study); and (3) incidence of IR-induced dicentric chromosomes was reduced in 53BP1-depleted cells (our study).

We found that the paired-foci frequency increased when Ku80, DNA-PKcs, or ATM was absent. Previous studies have shown that the frequency of CR was elevated in cells deficient in these factors, yet underlying mechanisms remain largely unknown^17^,^23^,^37^,^38^. Our findings can in part explain the elevated CR frequency in these deficient cells. Because paired foci indicate that two or more damaged chromatin regions are in close proximity, the incidence of DSB misrejoining may increase when the paired-foci frequency is elevated.

Among the C-NHEJ factors examined, Ku80 exhibited the greatest impact on foci pairing. A previous study showed that, *in vitro*, linear DNA formed loops in the presence of Ku, indicating that Ku tethers DNA ends^39^. Moreover, another study showed that ~10% of two intrachromosomal DSB ends were separated in Ku80-depleted cells, while almost none of the broken ends were separated in control cells^37^. The same study also showed that the mobility of DSB-containing chromatin was increased in Ku80-depleted cells^37^. Based on these previous studies, the increased foci pairing in Ku80^−/−^ cells observed in our study can be partly explained by the role of Ku80 (or the Ku70/80 heterodimer) in DSB end tethering and limiting of DSB movement.

Our results showed that knocking out DNA-PKcs had a milder effect on foci pairing than knocking out Ku80. Previous studies indicate that DNA-PKcs and other C-NHEJ factors (XLF, XRCC4, and ligase IV) also promote DSB end tethering^40^,^41^. However, it was shown that other C-NHEJ factors, such as XLF and XRCC4, can be recruited to DSB sites in the absence of DNA-PKcs, while Ku is required for the recruitment of all the other C-NHEJ factors to DSBs^42^,^43^,^44^. These relationships may explain the relatively small effect of DNA-PKcs on foci pairing. Our results indicate that the kinase activity of DNA-PKcs is not essential for the suppression of foci pairing, because chemical inhibition of DNA-PKcs did not affect the paired-foci frequency.

Unlike Ku80 and DNA-PKcs, XLF does not seem to suppress foci pairing, because paired foci did not increase in XLF-deficient cells. It is unknown whether XLF affects the recruitment of other C-NHEJ factors to DSBs, but it is likely that other C-NHEJ factors can assemble at DSBs without XLF, because DNA-PKcs, XRCC4, and ligase IV can interact directly with Ku^44^,^45^. Thus, despite the reported role of XLF in DSB end tethering, we consider XLF inessential for the suppression of foci pairing^41^.

In addition, our results suggest that relaxation of DSB-containing chromatin may increase the incidence of foci pairing. We found that foci pairing occurs more frequently in or at the periphery of HC than in EC. Several studies have shown that DSBs induce HC decondensation, indicating that the presence of DSBs can relax HC structure^8^,^46^. Furthermore, a study using heavy ion radiation showed that DSBs that occur within HC migrated to the periphery of HC^46^. These studies indicate that DSBs move with the relaxation of HC. DSB movement as HC relaxes, therefore, may increase the incidence of foci pairing. The role of chromatin relaxation in foci pairing is also suggested by the following results from our study: (i) depletion of 53BP1—a factor that contributes to relaxation of DSB-containing HC—reduced the frequency of paired foci and dicentric chromosomes; and (ii) the reduced foci pairing in 53BP1-depleted cells was recovered to the level in control cells when an HC building factor such as KAP-1 or CHD3 was concurrently depleted. DSB movement during chromatin relaxation probably also occurs in EC, albeit to a lesser extent than that in HC, because 53BP1 depletion did reduce foci pairing in EC. A recent study showed that mobility of IR-induced mCherry-BP1-2 foci was reduced in 53BP1^−/−^ MEFs^13^. According to the study, 53BP1 seems to promote mobility of foci in both EC and HC, because foci in these chromatin regions were not distinguished^13^. Taken together, results of our study suggest that 53BP1 can promote DSB mobility both in EC and HC through chromatin relaxation, and that 53BP1-dependent DSB movement may increase the incidence of foci pairing and CR.

Previous studies have shown that ATM also promotes HC relaxation, HC-DSB repair, mobility of unprotected telomeres, and joining between distal DSB ends during class switch recombination^12^,^47^,^48^,^49^. In addition, a recent study showed that ATM enhanced the mobility of IR-induced DSBs^50^. However, in contrast to 53BP1, ATM suppresses translocation frequency and foci pairing^23^,^24^. This discrepancy has yet to be reconciled; however, our study indicates that ATM kinase activity may be involved in suppression of foci pairing, because chemical inhibition of ATM increased the number of paired foci. Our results suggest that some ATM substrates may suppress foci pairing even in the presence of 53BP1. ATM substrates related to CR suppression will be investigated in a future study.

In the present study, we found and characterized foci-pairing events. Moreover, we obtained mechanistic insight into foci pairing by identifying several factors that affect this process. We believe that identification of additional factors that influence foci pairing will promote a better understanding of the molecular mechanisms of CR.

## Methods

### Cell culture and irradiation

hTERT-immortalized human fibroblasts (BJ-hTERT [normal]; 1BR-hTERT [normal]; 2BN-hTERT [XLF-deficient]; and AT5BI-hTERT [ATM-deficient]) were cultured in alpha-minimal essential medium (MEM) with 20% foetal bovine serum (FBS). Normal human primary fibroblasts (HE49) were cultured in MEM with 10% FBS. Wild-type (WT)#1, #2, Ku80^−/−^, and DNA-PKcs^−/−^ MEFs and NIH3T3 cells were cultured in Dulbecco’s modified Eagle’s medium/Ham’s F12 with 20% FBS. Irradiation was performed at room temperature in a ^137^Cs γ-ray irradiator at a dose rate of 1 Gy/min.

### Immunofluorescence staining and EdU detection

In this study, EdU was detected in all immunofluorescence experiments to allow identification of S phase cells. Cells were treated with 10 μM EdU for 30 min before fixation to label the S phase cells. At each time point after IR, the EdU-treated cells were washed once with phosphate-buffered saline (PBS) and fixed with 4% paraformaldehyde in PBS for 10 min at 4 °C, followed by permeabilization with 0.5% Triton X-100 in PBS for 5 min at 4 °C. Primary antibodies were then applied for 30 min at 37 °C, followed by reaction with Alexa 488- or 555-conjugated secondary antibodies (Life Technologies, USA) for 30 min at 37 °C. Nuclei were counterstained with 4′,6-diamidino-2-phenylindole (DAPI). All of the antibodies were diluted in 5% skim milk/Tris-buffered saline with 0.1% Tween 20. After immunofluorescence staining, EdU was detected following the manufacturer’s protocol. Images were acquired using a fluorescence microscope (DM6000B, Leica, Germany) or a confocal fluorescence microscope (LSM800, Zeiss, Germany). For image acquisition using DM6000B (Leica), x63 or x100 objective lenses, CCD camera (Orca R2, Hamamatsu Photonics), and FW4000 software were used. For image acquisition using LSM800 (Zeiss), x63 or x100 objective lenses, GaAsP detector, and ZEN 2.1 image acquisition software were used.

### Antibodies

The primary antibodies used were as follows: anti-p53-binding protein 1 (53BP1) (rabbit, Bethyl, USA, Cat #A300–272A); anti-phosphorylated histone H3 at serine 10 (phospho-H3) (mouse, clone 6G3, Cell Signaling Technology, USA, Cat #9706); anti-CENP-F (rabbit, Novus Biologicals, USA, Cat #NOV-NB500–101); anti-MRE11 (rabbit, clone 31H4, Cell Signaling Technology, USA, Cat #4847); anti-CtIP (rabbit, clone D76F7, Cell Signaling Technology, USA, Cat #9201); anti-phosphorylated histone H2AX at serine 139 (phospho-H2AX) (mouse, clone 2F3, BioLegend, USA, Cat #613402); anti-KAP-1 (rabbit, Bethyl, USA, Cat #A300-274A); anti-β-actin (mouse, clone 8H10D10, Cell Signaling Technology, USA, Cat #3700); anti-α/β-tubulin (rabbit, Cell Signaling Technology, USA, Cat #2148).

### Generation of cells expressing mAG-Geminin and mCherry-BP1-2

The lentiviral vector of monomeric Azami Green-tagged Geminin (mAG-Geminin) was kindly provided by Atsushi Miyawaki (RIKEN, Japan). The mAG-Geminin vector was lentivirally introduced into BJ-hTERT cells, and the cells were cultured in the presence of 200 μg/mL Zeocin for 14 days to obtain vector-integrated clones. Clones that expressed mAG-Geminin were selected visually under a fluorescence microscope. The mCherry-BP1-2 pLPC-Puro vector was a kind gift from Titia de Lange (Rockfeller University, USA) via Addgene (plasmid #19835). This vector encodes a fusion protein of mCherry and the minimal focus-forming region (amino acids 1220–1711) of human 53BP1. The vector was introduced into BJ-hTERT or NIH3T3 cells by electroporation (Neon, Life Technologies, USA), and the cells were cultured in the presence of 2 μg/mL puromycin for 14 days to obtain vector-integrated clones. Clones that formed discrete nuclear foci of mCherry-BP1-2 after γ-irradiation were selected and used in the experiments. The BJ-hTERT clone that expressed both mCherry-BP1-2 and mAG-Geminin was obtained by introducing the mCherry-BP1-2 pLPC-Puro vector into mAG-Geminin-expressing BJ-hTERT cells by electroporation and subsequently selecting on puromycin, as described above.

### Live cell imaging

Live cell images were obtained using a BioStation cell observation system (Nikon, Japan). BJ-hTERT cells that expressed both mCherry-BP1-2 and mAG-Geminin were plated in glass bottom dishes for 2–3 days before image acquisition. On the day of live cell imaging, the cells were irradiated with 4 Gy γ-rays and placed immediately in a humidified chamber with 5% CO_2_, where they were allowed to stand for 1–2 h until the temperature in the chamber stabilized. The live cell images were then captured beginning at 2 h after IR at 5-min intervals using a 40× objective. Capture of live cell images began 2 h after IR because the BioStation requires 1–2 h to stabilise the temperature in the chamber. In total, 10 fields were captured at each time point. The images were stored as AVI and TIFF files, and foci pairing was evaluated using Photoshop Element 9 (Adobe Systems, USA).

### Analysis of foci pairing

Paired foci of 53BP1, mCherry-BP1-2, or phospho-H2AX were defined as two or more neighbouring foci as shown in Fig. 1a. For analyses using fixed cells, foci pairing was evaluated by eye under a fluorescence microscope. The G1 phase cells were identified as those negative for EdU (an S phase marker) and phospho-H3 (a G2/M phase marker) or CENP-F (an S/G2 phase marker). In all of the experiments using fixed cells (except for the one with results shown in Fig. 7b), foci pairing was examined in 50 cells for each sample or at each time point in two independent experiments; that is, a total of 100 cells were examined for each sample or at each time point. For the experimental results shown in Fig. 7b, foci pairing was examined in >30 cells for each sample in two independent experiments; that is, a total of >60 cells were examined for each sample. For living cells, foci pairing was evaluated by visual inspection of the captured images. Among these cells, G1 phase cells were identified as those negative for mAG-Geminin (an S/G2/M phase marker). Images showing the three-dimensional pairing of foci were obtained with an Applied Precision DeltaVision OMX microscope (GE Life Sciences, USA) using the settings for conventional image capture with a 60× objective lens. A series comprising 16 images was acquired along the z-axis (0.25-μm intervals over 4 μm), with images stacked into a single-layer image by deconvolution. The deconvoluted z-stack images were converted into a three-dimensional rendering of a nucleus containing the 53BP1 foci. A three-dimensional rendering of a single nucleus and polygon imaging were performed using Imaris 8.0.1 (Zeiss, Germany).

### siRNA transfection

siRNA transfection was performed using Lipofectamine RNAi MAX (ThermoFisher Scientific, USA) according to the manufacturer’s protocol. Briefly, 1.4~2 × 10^5^ cells were plated in a 35-mm dish with siRNA/Lipofectamine RNAi MAX in Opti-MEM I (ThermoFisher Scientific, USA). The medium was changed at 8–16 h after siRNA transfection, and the cells were cultured for 2 additional days before experiments. The final concentration of all siRNAs was 30 nM. The siRNA sequences used in the present study are listed in Supplementary Figure S13. Note that the 53BP1 siRNAs used in this study do not interfere with the expression of mCherry-BP1-2, because the mRNA sequence of mCherry-BP1-2 does not contain the target sequences of the siRNAs. The control siRNA used in this study was AllStars Negative Control siRNA (Qiagen, Germany).

### Establishment of short hairpin RNA-inducible cells

We used the pTRIPZ inducible lentiviral shRNAmir system (GE Life Sciences, USA) to deplete 53BP1 or MDC1 in confluent BJ-hTERT cells used in the chromosome experiments. In this system, the shRNA constructs were expressed as human microRNA-30 primary transcripts in the presence of doxycycline (see the manufacturer’s website for details). pTRIPZ vectors that target 53BP1 or MDC1 and the nontargeting control pTRIPZ vector were obtained from the manufacturer. The pTRIPZ vector was introduced lentivirally into BJ-hTERT cells, and then the cells were cultured in the presence of 15 μg/mL puromycin to obtain vector-integrated cells. Because the efficiency of vector integration was very high, we used the bulk of the puromycin-resistant cells, rather than a clone, in the chromosome experiments.

### Chemical inhibitors

The DNA-PK inhibitor (NU7441, Tocris Bioscience, UK), ATM inhibitor (KU55933, Calbiochem, Germany), and Chk1/2 inhibitor (SB218078, Tocris Bioscience, UK) were used to treat cells at final concentrations of 10, 10, and 2.5 μM, respectively. The efficacy of these inhibitors, including in the inhibition of cell cycle checkpoints and/or DSB repair, is well established^19^,^51^,^52^.

### Statistical analysis

Statistical analyses were performed using Prism 6 (GraphPad Software, USA).

## Additional Information

**How to cite this article**: Yamauchi, M. *et al*. Regulation of pairing between broken DNA-containing chromatin regions by Ku80, DNA-PKcs, ATM, and 53BP1. *Sci. Rep.* **7**, 41812; doi: 10.1038/srep41812 (2017).

**Publisher's note:** Springer Nature remains neutral with regard to jurisdictional claims in published maps and institutional affiliations.

## Supplementary Material

Supplementary Information

Supplementary Movie S1
