# Supplementary material for: Regulation of pairing between broken DNA-containing chromatin regions by Ku80, DNA-PKcs, ATM, and 53BP1
Source: Sci Rep. 2017 Feb 3;7:41812. doi: 10.1038/srep41812 (PMC5290537; doi:10.1038/srep41812)
Supplement: Supplementary Information [file srep41812-s1.pdf]

Supplementary information

Regulation of pairing between broken DNA-containing chromatin regions
by Ku80, DNA-PKcs, ATM, and 53BP1

Motohiro Yamauchi[1]*, Atsushi Shibata[2], Keiji Suzuki[3], Masatoshi Suzuki[4], Atsuko Niimi[5], Hisayoshi Kondo[6], Miwa Miura[7], Miyako Hirakawa[1], Keiko Tsujita[8], Shunichi Yamashita[3], Naoki Matsuda[1]

[1]Department of Radiation Biology and Protection, Atomic Bomb Disease Institute, Nagasaki University, 1-12-4 Sakamoto, Nagasaki, 852-8523, Japan
[2]Advanced Scientific Research Leaders Development Unit, Gunma University, 3-39-22 Showa-machi, Maebashi, Gunma, 371-8511, Japan
[3]Department of Radiation Medical Sciences, Atomic Bomb Disease Institute, Nagasaki University, 1-12-4 Sakamoto, Nagasaki, 852-8523, Japan
[4]Department of Pathology, Institute of Development, Aging and Cancer, Tohoku University, 4-1 Seiryo-machi, Aoba-ku Sendai, Miyagi, 980-8575, Japan
[5]Gunma University Initiative for Advanced Research, 3-39-22 Showa-machi, Maebashi, Gunma, 371-8511, Japan
[6]Department of Global Health, Medicine and Welfare, Atomic Bomb Disease Institute, Nagasaki University, 1-12-4 Sakamoto, Nagasaki, 852-8523, Japan
[7]Radioisotope Center, Center for Frontier Life Sciences, Nagasaki University, 1-12-4 Sakamoto, Nagasaki, 852-8523, Japan
[8]School of Medicine, Nagasaki University, 1-12-4 Sakamoto, Nagasaki, 852-8523, Japan
*To whom correspondence should be addressed.
Tel: +81-95-819-7164; Fax: +81-95-819-7153; Email: motoyama@nagasaki-u.ac.jp

Legends for Supplementary Figures and Tables

Figure S1 – Colocalisation of 53BP1 foci with phospho-H2AX foci.

BJ-hTERT cells were irradiated with 2 Gy ionizing radiation (IR) and fixed at indicated time points. The fixed cells were then subjected to immunofluorescence staining for 53BP1 and serine 139-phosphorylated histone H2AX. Nuclei were counterstained with 4′,6-diamidino-2-phenylindole (DAPI). White arrowheads represent paired foci.

Figure S2 – The cell cycle phase in which dicentric chromosomes are produced.

(a) HE49 cells (normal human primary fibroblasts) in the G0/G1, S, or G2 phase were irradiated with 2 Gy IR, and cells that progressed to mitosis were harvested to prepare chromosome samples. To analyse the chromosomes of cells irradiated in the G0/G1 phase, confluent cells were irradiated and replated at low density to allow the cell cycle to progress, and then mitotic cells were harvested at 48 h after IR. To analyse chromosomes of cells irradiated in the S phase, S phase cells were pulse-labelled with 10 μM bromodeoxyuridine (BrdU) for 30 min and then irradiated. Mitotic cells were harvested at 10 h after exposure to IR. To analyse chromosomes of cells irradiated in the G2 phase, cells were irradiated and mitotic cells were harvested at 4 h after exposure to IR. To avoid bias by IR-induced cell cycle checkpoints, the experiments were performed in the presence of an ATM inhibitor for G0/G1 phase irradiated samples, and both an ATM inhibitor and Chk1/2 inhibitor were used for samples irradiated in the S or G2 phase. Chromosomes of G0/G1 and G2 phase-irradiated cells were stained with Giemsa solution. S phase-irradiated cells were subjected to BrdU staining and centromere/telomere fluorescence *in situ* hybridization. Only BrdU (+) chromosomes were analysed.

(b) Typical immunofluorescence images of 53BP1 foci (red), EdU (green), and phospho-H3 (green) in BJ-hTERT cells exposed to IR. The nuclei were counterstained with 4′,6-diamidino-2-phenylindole (DAPI, blue).

Figure S3 – Analysis of 53BP1 and mCherry-BP1-2 foci in the G1 phase.

(a) Frequency of paired 53BP1 foci in paired foci(+) BJ-hTERT cells (G1 phase).

(b) Kinetics of the number of 53BP1 foci in total cells and paired foci(+) cells (BJ-hTERT, G1 phase).

(c) Frequency of paired 53BP1 foci in paired foci(+) BJ-hTERT cells (G1 phase) after exposure to varying doses of IR. Results at 8 h after IR are shown.
(d) The number of 53BP1 foci in total cells and paired foci(+) cells after exposure to varying doses of IR (BJ-hTERT, G1 phase). Results at 8 h after IR are shown.
(e) Frequency of paired 53BP1 foci in total cells including paired foci(–) cells after exposure to varying doses of IR (BJ-hTERT, G1 phase). Results at 8 h after IR are shown.
(f) Kinetics of the number of the total and paired 53BP1 foci after IR in BJ-hTERT cells (G1 phase).
(g) Kinetics of the number of the total and paired mCherry-BP1-2 foci after IR in BJ-hTERT cells (G1 phase).
(h) Frequency of paired mCherry-BP1-2 foci in fixed BJ-hTERT cells (G1 phase). Plotted in Supplementary Figure S3a, c, e, and h are the percentages of paired foci among the total foci in individual cells. Red bars represent means. The statistical comparison in Supplementary Figure S3a, c, e, and h was performed using the Dunn's multiple comparison test (alpha=0.05). Results shown in Supplementary Figure S3f and g represent the mean ± SD based on two independent experiments. IR, ionizing radiation; 53BP1, p53-binding protein 1.

Figure S4 – Examples of foci pairing in live cells.

(a) An example of dynamic pairing of mCherry-BP1-2 foci in a living BJ-hTERT cell. The image sequence shows mCherry-BP1-2 foci (red) in a G1 cell. The numbers at the top right indicate the time after ionizing radiation (hr:min). The white arrowheads indicate two foci undergoing dynamic pairing to become a single focus (enlarged in the inset).
(b) Another example of dynamic pairing of mCherry-BP1-2 foci. The white arrowheads indicate four foci undergoing dynamic pairing (enlarged in the inset).
(c) An example of static pairing of mCherry-BP1-2 foci in a living BJ-hTERT cell. The white arrowheads indicate two foci pairing continuously (enlarged in the inset).

Figure S5 – Foci number kinetics and paired-foci frequency after ionizing radiation.

(a) Kinetics of the number of the 53BP1 foci in Ku80$^{-/-}$ MEFs, DNA-PKcs$^{-/-}$ MEFs and two WT MEFs (WT#1 and #2) after IR (G1 phase).

(b) Effect of DNA-PK inhibitor on the number of phospho-H2AX foci. DNA-PK inhibitor (NU7441, 10 μM) or vehicle (dimethyl sulphoxide, DMSO) was applied 30 min before IR exposure until the time of fixation (0.5 h or 24 h after 2 Gy of IR).

(c) Kinetics of the number of 53BP1 foci in BJ-hTERT (control) and 2BN-hTERT (XLF-deficient) cells after IR (G1 phase).

(d) Frequency of paired 53BP1 foci in BJ-hTERT and 2BN-hTERT cells (G1 phase) after 2 Gy IR. Plotted are the percentages of paired foci among the total foci in individual cells. Statistical analyses were performed using the two-tailed Mann-Whitney test (alpha=0.05). Red bars represent means.

(e) Kinetics of the number of 53BP1 foci in BJ-hTERT (control) and AT5BI-hTERT (ATM-deficient) cells after IR (G1 phase). Results shown in Supplementary Figure S5a–c and e represent the mean ± SD of two independent experiments. IR, ionizing radiation; 53BP1, p53-binding protein 1; MEF, mouse embryonic fibroblast; WT, wild-type.

Figure S6 Chromosome analysis and foci number kinetics

(a) Fluorescence *in situ* hybridization (FISH) of centromeres (red) and telomeres (green). The white arrowhead indicates a chromosome break. Chromosomes were counterstained with DAPI (blue).

(b) Percentage of chromosome break(+) BJ-hTERT cells depleted of 53BP1 or MDC1. Same chromosome samples as used in Figure 6b experiments were analysed. A total of 100 metaphase cells was analysed by centromere/telomere FISH.

(c) Frequency of IR-induced chromosomes in BJ-hTERT cells depleted of 53BP1 or MDC1. Same chromosome samples as used in Figure 6b experiments were analysed. A total of 100 metaphase cells was analysed by centromere/telomere FISH.

(d) Kinetics of the number of mCherry-BP1-2 foci in BJ-hTERT cells treated with 53BP1 siRNAs. The result represents the mean ± SD based on two independent experiments.

(e) Typical images of mCherry-BP1-2 foci in BJ-hTERT cells treated with 53BP1 siRNAs.

Figure S7 – Full-length images of western blots shown in Figure 4b and d.

(a) A full-length blot of MRE11 shown in Figure 4b.

(b) A full-length blot of β-actin shown in Figure 4b.

(c) A full-length blot of CtIP shown in Figure 4d

(d) A full-length blot of β-actin shown in Figure 4d

Figure S8 – Full-length images of western blots shown in Figure 6c and d.

(a) A full-length blot of 53BP1 shown in Figure 6c.

(b) A full-length blot of MDC1 shown in Figure 6d.

(c) A full-length blot of α/β-tubulin shown in Figure 6c and d.

Figure S9 – Full-length images of western blots shown in Figure 6e.

(a) A full-length blot of 53BP1 shown in Figure 6e.

(b) A full-length blot of β-actin shown in Figure 6e.

Figure S10 – Full-length images of western blots shown in Figure 7c.

(a) A full-length blot of 53BP1 shown in Figure 7c.

(b) A full-length blot of β-actin shown in Figure 7c.

Figure S11 – Full-length images of western blots of 53BP1 and β-actin.

Depletion of 53BP1 performed in Figure 7d and e experiments was confirmed.

Figure S12 – Full-length images of western blots of KAP-1 and β-actin.

Depletion of KAP-1 performed in Figure 7d and e experiments was confirmed.

Figure S13 – siRNA sequences

Sequences of siRNAs used in this study are listed.

Table S1 – Paired-foci frequency in BJ-hTERT cells.

Shown are the sums of total and paired 53BP1 foci in all cells analysed. The percentage of paired foci in each sample was calculated based on the sums of total and paired foci.

Table S2 – Paired-foci frequency in wild-type and Ku80$^{-/-}$ cells.

Shown are the sums of total and paired 53BP1 foci in all cells analysed. The percentage of paired foci in each sample was calculated based on the sums of total and paired foci. WT, wild type.

Table S3 – Paired-foci frequency in wild-type and DNA-PKcs$^{-/-}$ cells.

Shown are the sums of total and paired 53BP1 foci in all cells analysed. The percentage of paired foci in each sample was calculated based on the sums of total and paired foci. WT, wild type.

Table S4 – Paired-foci frequency in 2BN-hTERT cells.

Shown are the sums of total and paired 53BP1 foci in all cells analysed. The percentage of paired foci in each sample was calculated based on the sums of total and paired foci.

Table S5 – Paired-foci frequency in BJ-hTERT cells depleted of MRE11 or CtIP.

Shown are the sums of total and paired 53BP1 foci in all cells analysed. The percentage of paired foci in each sample was calculated based on the sums of total and paired foci.

Table S6 – Paired-foci frequency in AT5BI-hTERT cells.

Shown are the sums of total and paired 53BP1 foci in all cells analysed. The percentage of paired foci in each sample was calculated based on the sums of total and paired foci.

Table S7 – Paired-foci frequency in BJ-hTERT cells depleted of 53BP1.

Shown are the sums of total and paired mCherry-BP1-2 foci in all cells analysed. The percentage of paired foci in each sample was calculated based on the sums of total and paired foci.

Table S8 – Paired-foci frequency in euchromatin and heterochromatin in NIH3T3 cells.

Shown are the sums of total and paired mCherry-BP1-2 foci in euchromatin (EC) or heterochromatin (HC) in all cells analysed. The percentage of paired foci in each sample was calculated based on the sums of total and paired foci.

Table S9 – Paired-foci frequency in BJ-hTERT cells depleted of 53BP1 alone or both 53BP1 and heterochromatin-building factors.

Shown are the sums of total and paired mCherry-BP1-2 foci in all cells analysed. The percentage of paired foci in each sample was calculated based on the sums of total and

paired foci.

Supplementary movie

Movie S1. A movie of three-dimensional deconvoluted images of paired 53BP1 foci. A three-dimensional foci image was obtained as described in Materials and Methods, and the movie was produced using Imaris 8.0.1 (Zeiss, Germany).

Supplementary Methods

Detection of dicentric chromosomes by centromere/telomere fluorescence in situ hybridization

Previously, we found that cell cycle checkpoints affect the frequency of IR-induced chromosome translocation and dicentric chromosomes[1]. To avoid a checkpoint bias in determining the frequency of dicentric chromosomes, mitotic chromosomes were harvested in the presence of p53 shRNA and the Chk1/2 inhibitor. The lentiviral vector that encodes p53 shRNA (shp53 pLKO.1 Puro) was a kind gift from Bob Weinberg (Addgene plasmid #19119)[2]. Before the chromosome experiments, p53 shRNA was introduced lentivirally into 53BP1-, MDC1-, or control shRNA-inducible BJ-hTERT cells. The cells were then grown to confluence to synchronize the cell cycle in G0/G1. Next, doxycycline (1 μg/ml) was applied for at least 3 days to induce 53BP1 or MDC1 shRNA. The cells (depleted of p53 and 53BP1 or MDC1) were then irradiated with 2 Gy γ-rays and replated immediately at low density in medium containing the Chk1/2 inhibitor (2.5 μM) to allow the cell cycle to progress to mitosis. Colcemid (0.025 μg/ml) was added at 24 h after replating to allow mitotic cells to accumulate. At 40 h after replating, the mitotic cells were shaken from the culture flasks and suspended in foetal bovine serum-containing medium. The cells were washed once with phosphate-buffered saline (PBS) and subjected to hypotonic treatment in 0.075 M KCl for 20 min at room temperature. The cells were fixed with Carnoy's fixative (methanol:acetic acid = 3:1) for 30 min at 4°C. After fixation, the cell suspension in the fixative was dropped onto a glass slide and dried overnight. The dried glass slide was washed briefly in PBS, immersed in 4% formalin in PBS for 2 min at room temperature, and then washed three times with PBS. Peptide nucleic acid probes for centromeres and telomeres (Panagene, Korea) were applied to the cells, and the glass slide was heated for 3 min at 80°C to denature the DNA, followed by hybridization in a dark box for 2 h at room temperature. The slide was then washed twice in wash buffer I (70% formamide in TE buffer) and twice with wash buffer II (TE buffer containing 0.15 M NaCl and 0.05% Tween 20). The washed slide was briefly air dried and counterstained with SlowFade Antifade Reagent containing DAPI (Life Technologies, USA).

Western blotting

Except for Figure 6c, d, and Supplementary Figure S8, western blotting was performed as follows: Cells grown in a 35 mm dish were washed once with PBS and were lysed with 30–100 μl of 2x Laemli's sample buffer (Sigma Aldrich, USA). The amount of sample buffer was changed depending on density of cells. Then, the cell lysates were boiled for 5 min at 95°C, followed by sonication for 2 min at room temperature (r.t.). Ten microliter of the cell lysates were applied to wells in a 7.5% SDS-PAGE gel (Atto, Japan) and proteins were separated by SDS-PAGE. Molecular weight marker (ECL Plex Fluorescent Rainbow Markers, GE Healthcare, USA) was also applied to the gel. After SDS-PAGE, the gel was shaken in Tris/glycine buffer for 15–20 min and proteins were transferred to PVDF membrane for fluorescence western blot (Immobilon FL, Merck-Millipore) for 1 h. Then, the membrane was incubated with blocking solution (EzBlock BSA, Atto, Japan) for 1 h at r.t. After blocking, primary antibodies against a target protein and a loading control protein (β-actin or α/β-tubulin) were concomitantly treated overnight (12–18 h) at 4°C. After washing (5 min x 3) with Tris-buffered saline/1% Tween 20 (TBS-T), secondary antibodies conjugated with Alexa 555 and Alexa 647 were concomitantly treated for 1 h at r.t. All antibodies were diluted in TBS-T. Dilution ratio is: 1:200 for primary antibodies against target proteins; 1:1000 for primary antibodies against loading control proteins. After washing (5 min x 3) with TBS-T, the membrane was air dried. The primary antibodies used for western blotting were as follows: anti-p53-binding protein 1 (53BP1) (rabbit, Bethyl, USA, Cat #A300-272A); anti-MRE11 (rabbit, clone 31H4, Cell Signaling Technology, USA, Cat #4847); anti-CtIP (rabbit, clone D76F7, Cell Signaling Technology, USA, Cat #9201); anti-KAP-1 (rabbit, Bethyl, USA, Cat #A300-274A); anti-β-actin (mouse, clone 8H10D10, Cell Signaling Technology, USA, Cat #3700); anti-α/β-tubulin (rabbit, Cell Signaling Technology, USA, Cat #2148). Protein bands were visualized by FLA-5100 (Fuji film, Japan) using 532 nm (for Alexa 555) or 635 nm (for Alexa 647) laser. Voltage of photomultiplier was changed according to expression level of proteins so that band intensity does not saturate. Blot images were acquired using image acquisition software (Image Reader FLA-5000 Series ver.1.0, Fuji film, Japan). Acquired images were exported as TIFF files using Multi Gauge ver.3.0 (Fuji film, Japan).

For western blotting shown in Figure 6c, d, and Supplementary Figure S8, cells

grown in a T75 flask were once washed with PBS and lysed with RIPA buffer (pH7.2, 50 mM Tris-HCl, 150 mM NaCl, 1% NP-40, 1% Sodium Deoxycholate, 0.1% SDS). Then, protein concentration was measured by BCA protein assay kit (ThermoFisher Scientific, USA). After mixing with 2x Laemli's sample buffer (Sigma Aldrich, USA), 16 μg of proteins were applied to wells in a SDS-PAGE gel and proteins were separated by SDS-PAGE. Molecular weight marker (Full Range Rainbow Molecular Weight Markers, GE Healthcare, USA) was also applied to the gel. After SDS-PAGE, the gel was shaken in Tris/glycine buffer for 30 min and proteins were transferred to PVDF membrane (Immobilon, Merck-Millipore) for 1 h. Then, the membrane was blocked with 10% skim milk/TBS-T overnight at r.t. After blocking, a primary antibody against 53BP1, MDC1, or α/β-tubulin was treated for 2 h at r.t. After washing (5 min x 3) with TBS-T, a biotinylated secondary antibody (GE Healthcare, USA) was treated for 1 h at r.t. After washing (5 min x 3) with TBS-T, streptavidin-conjugated alkaline phosphatase (BioLegend, USA) was treated for 2 h at r.t. After washing (5 min x 3) with TBS-T, protein bands were visualized by adding NBT/BCIP (Roche, Switzerland) diluted in NBT solution (pH9.5, 50 mM Tris, 50 mM NaCl, 25 mM $MgCl_2 \cdot 6H_2O$).

Supplementary References

1 Yamauchi, M. *et al*. Mode of ATM-dependent suppression of chromosome translocation. *Biochem. Biophys. Res. Commun.* **416**, 111–118, doi:10.1016/j.bbrc.2011.11.006 (2011).

2 Godar, S. *et al*. Growth-inhibitory and tumor- suppressive functions of p53 depend on its repression of CD44 expression. *Cell* **134**, 62–73, doi:10.1016/j.cell.2008.06.006 (2008).

Supplementary Figure S1

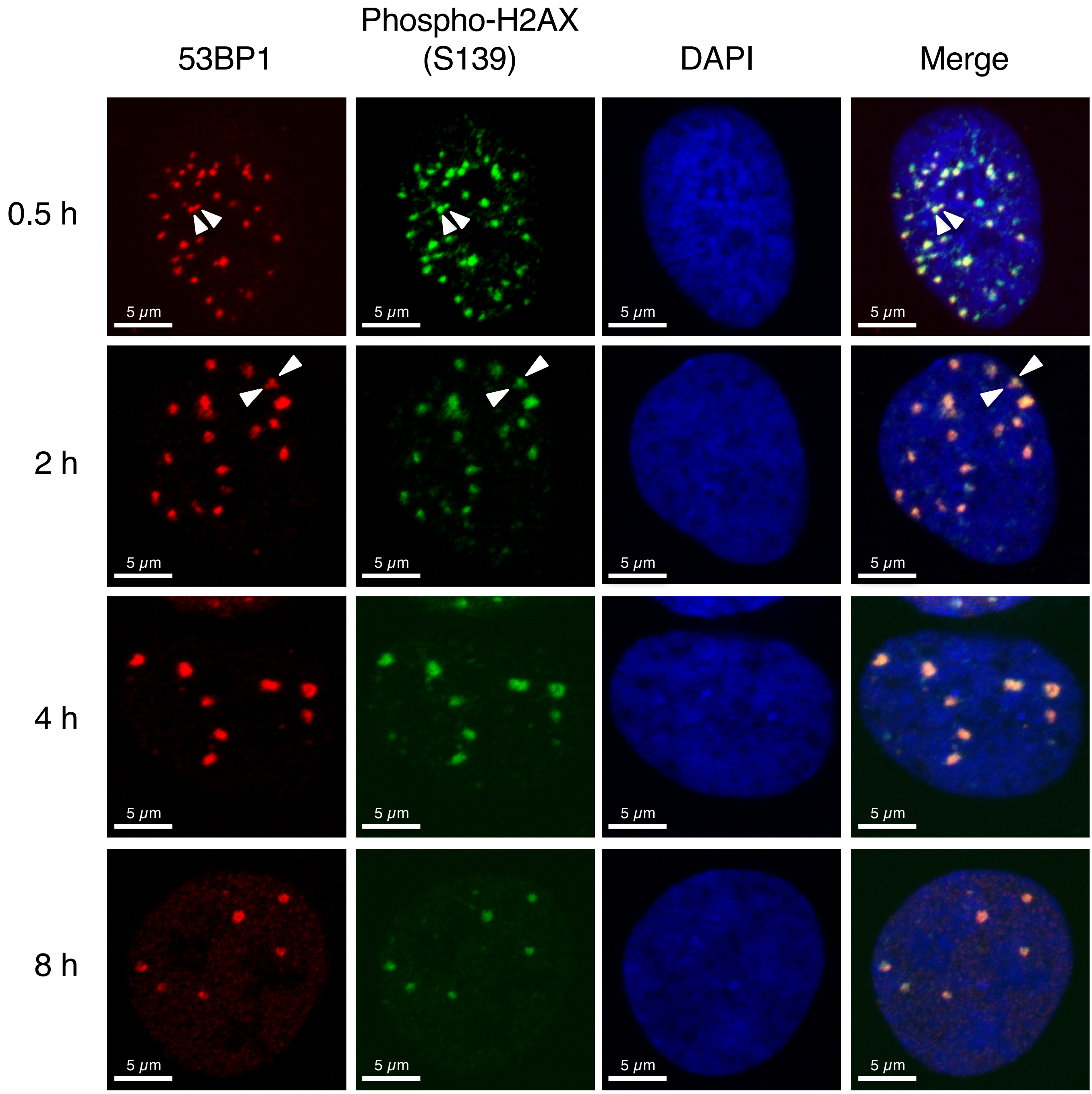

Supplementary Figure S2

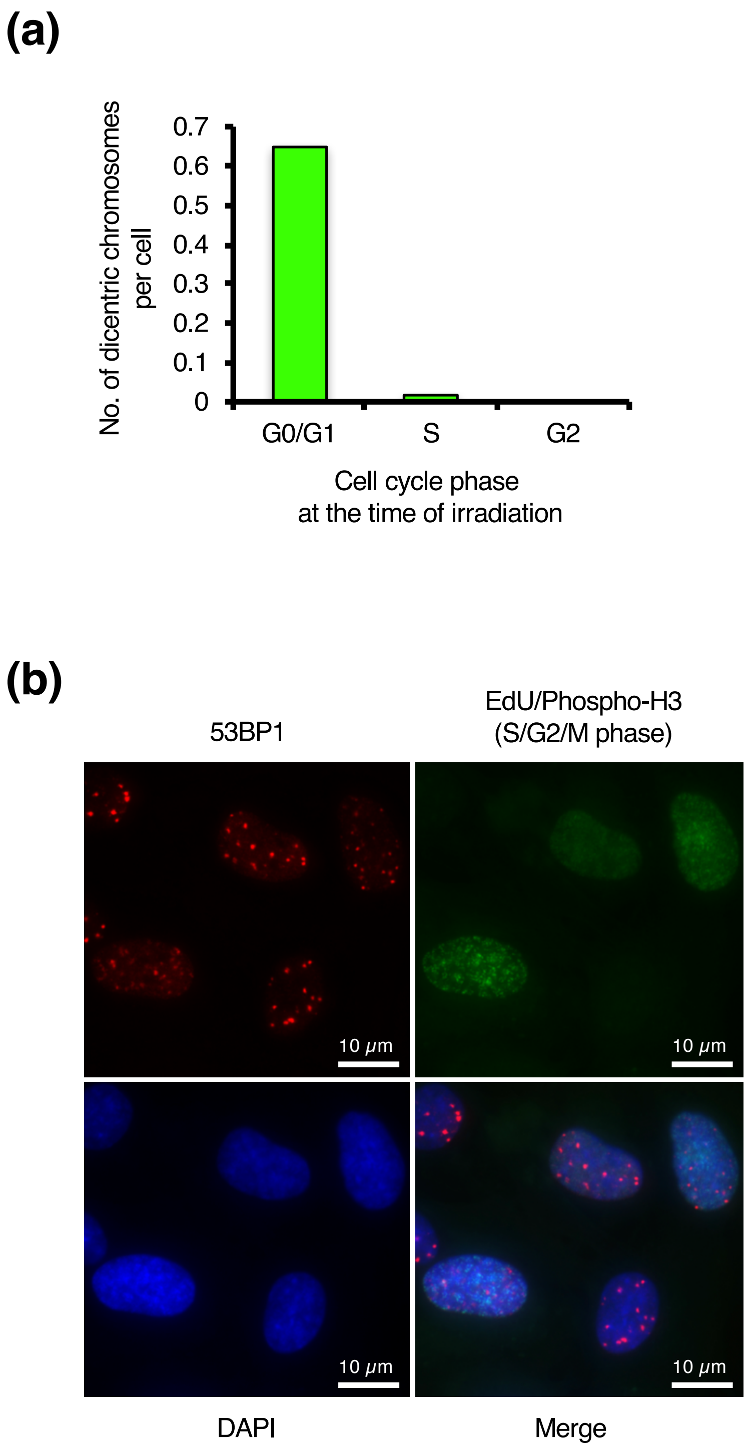

Supplementary Figure S3

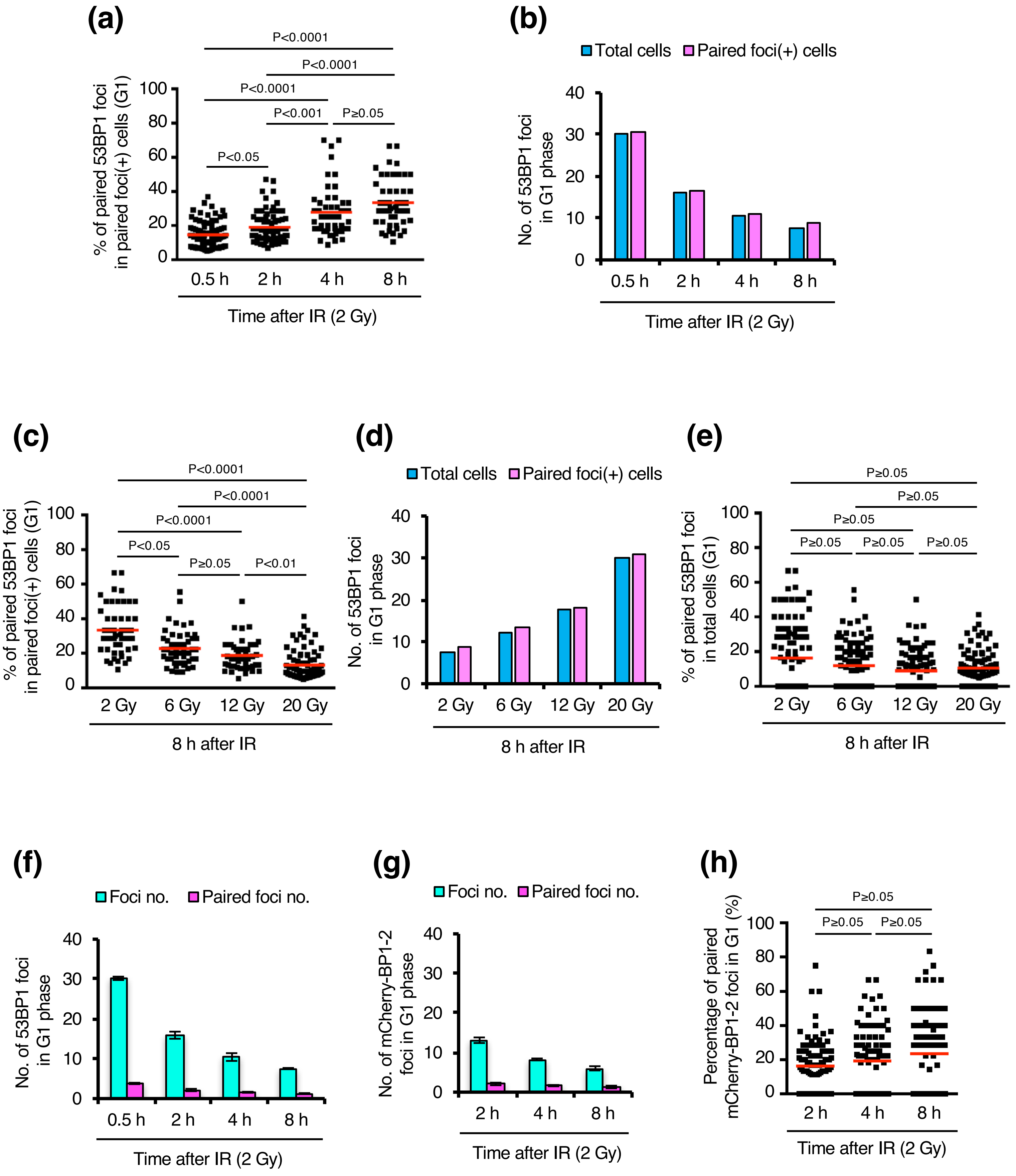

Supplementary Figure S4

(a)

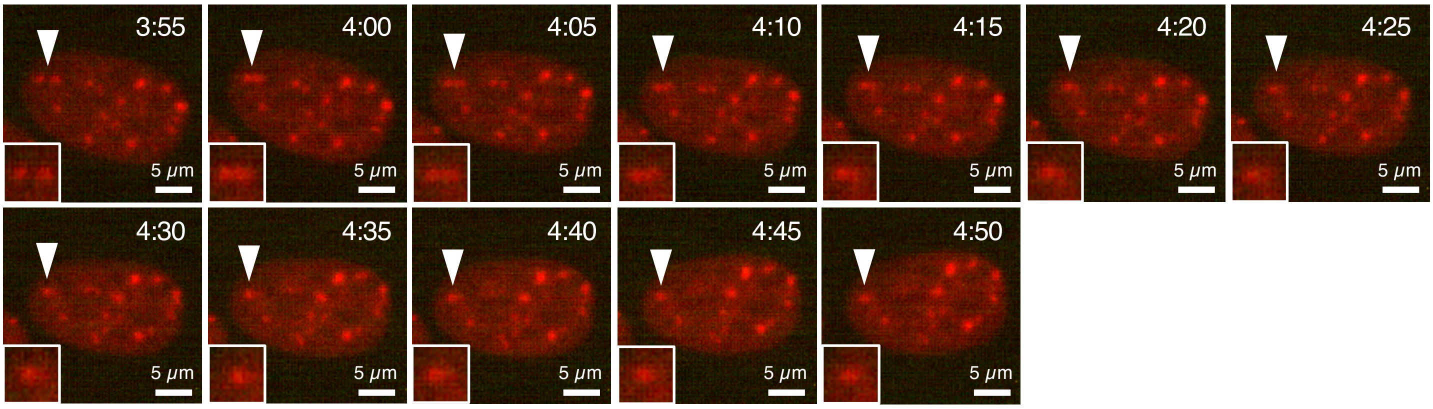

(b)

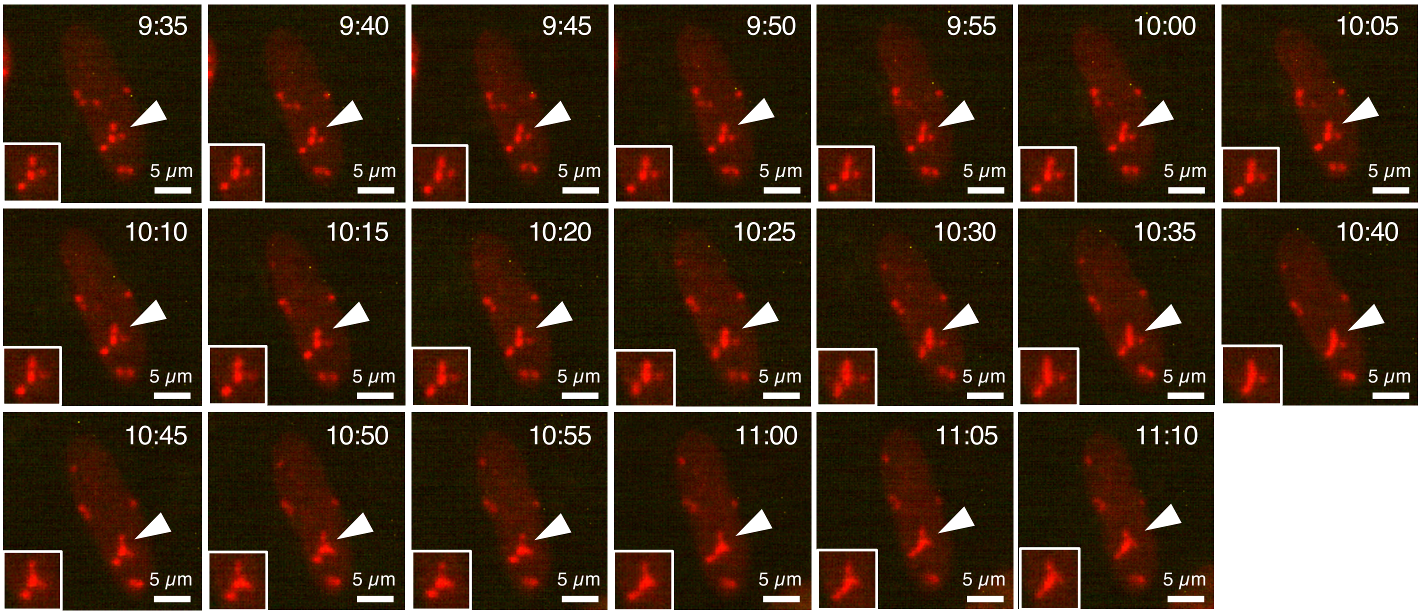

(c)

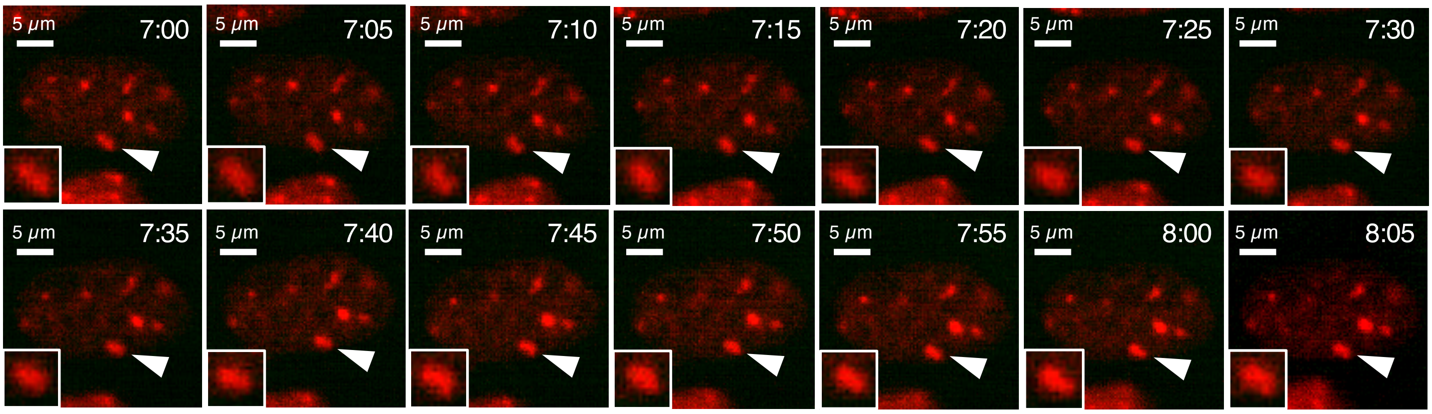

Supplementary Figure S5

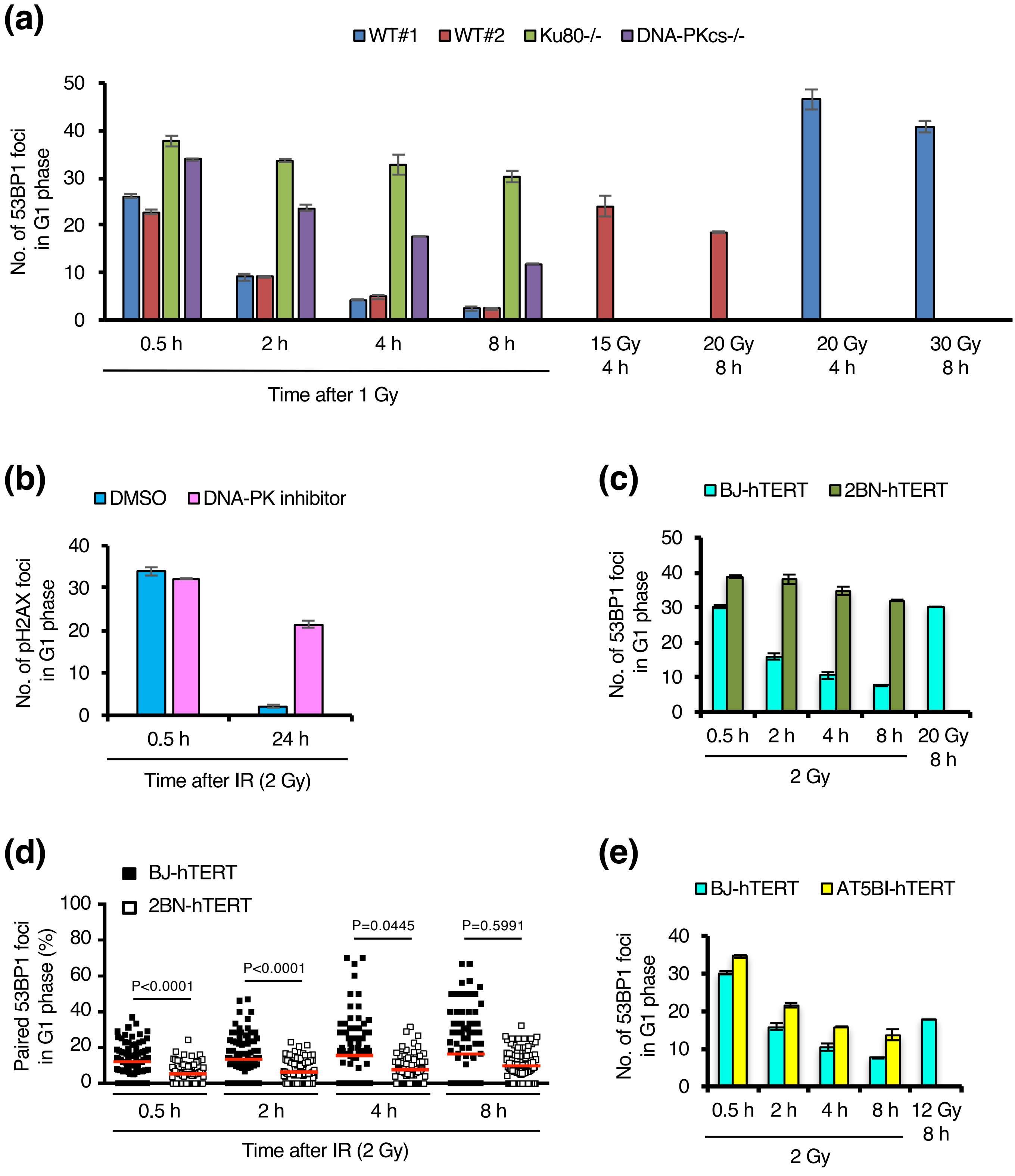

Supplementary Figure S6

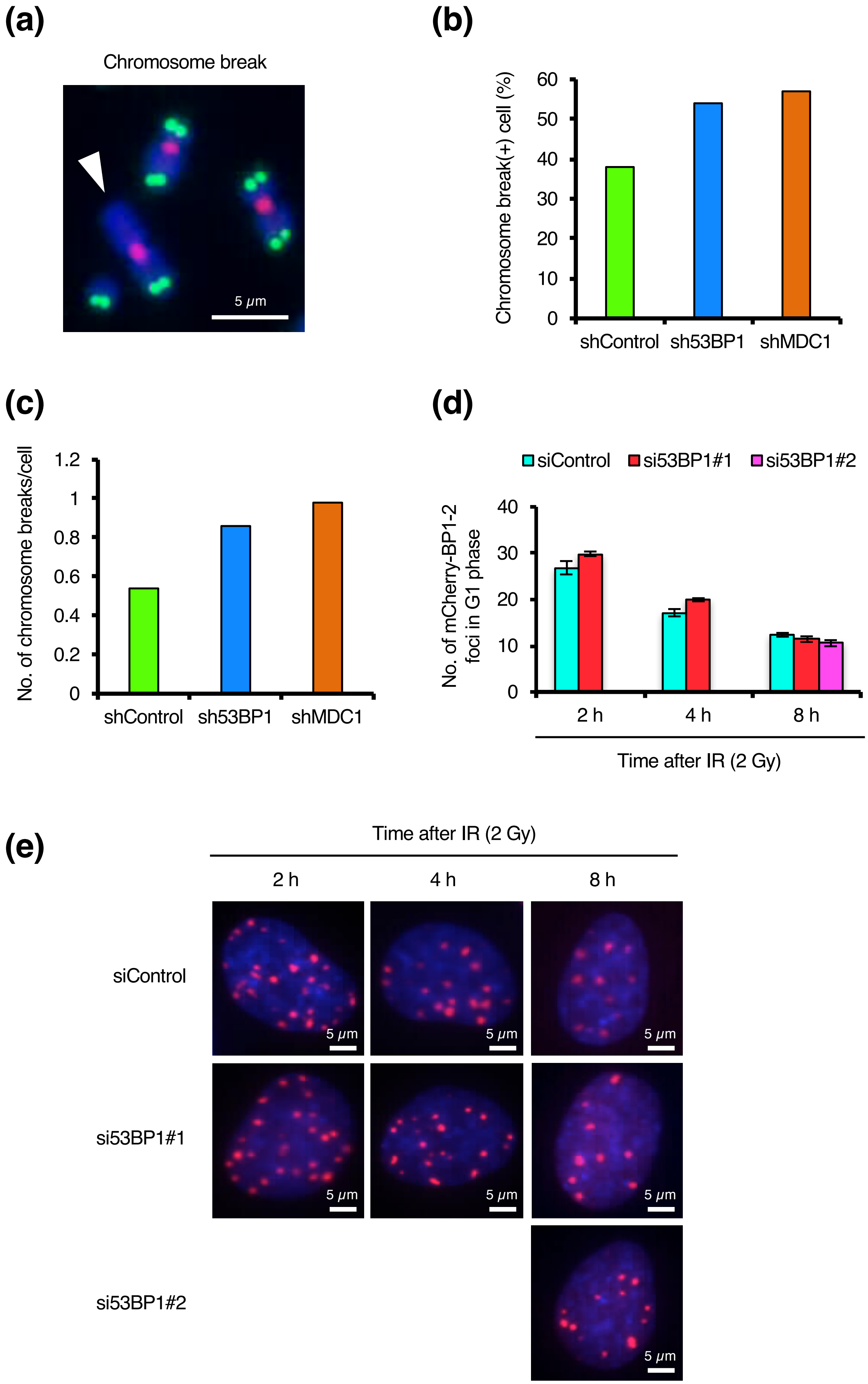

Supplementary Figure S7

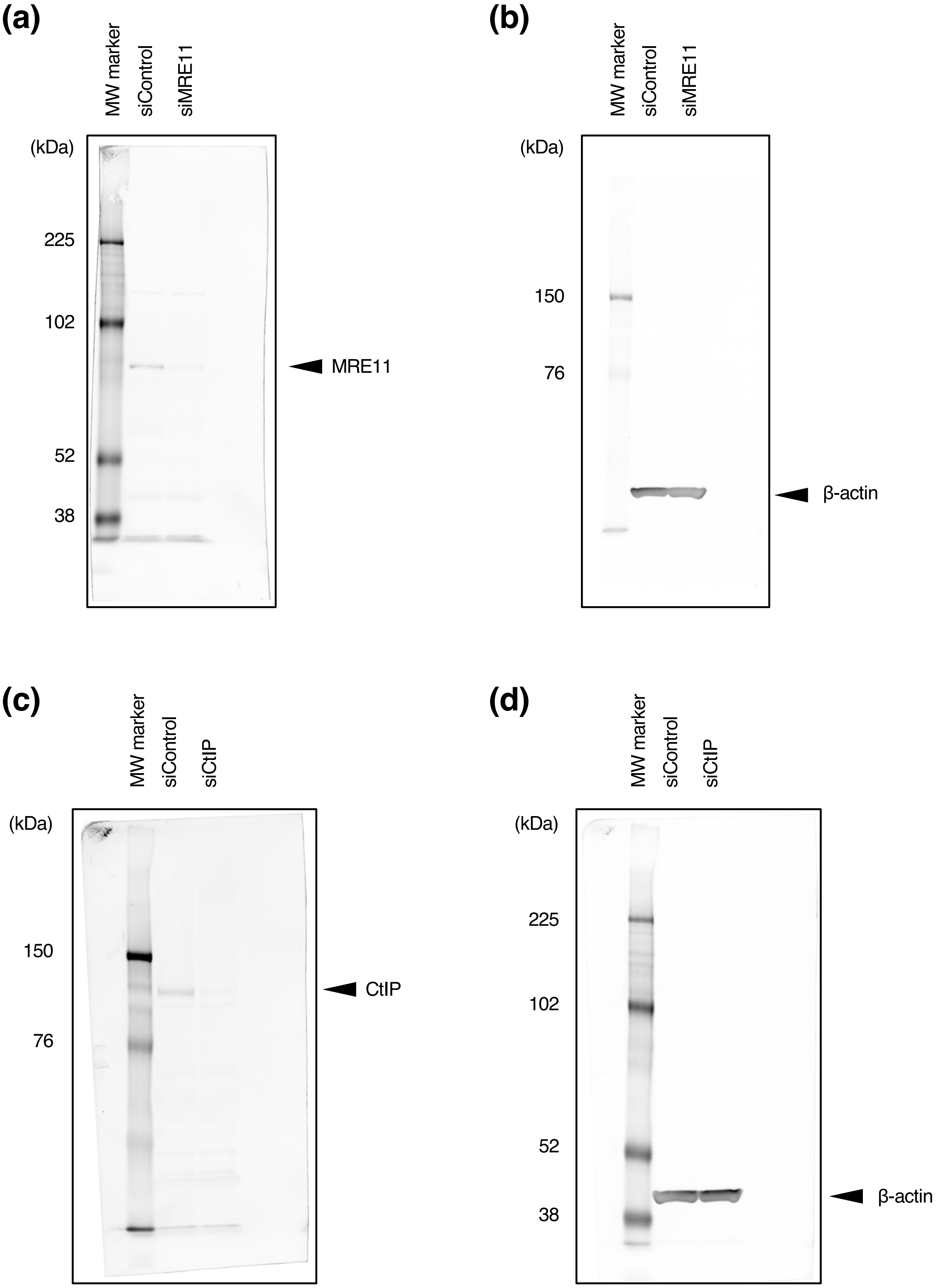

Supplementary Figure S8

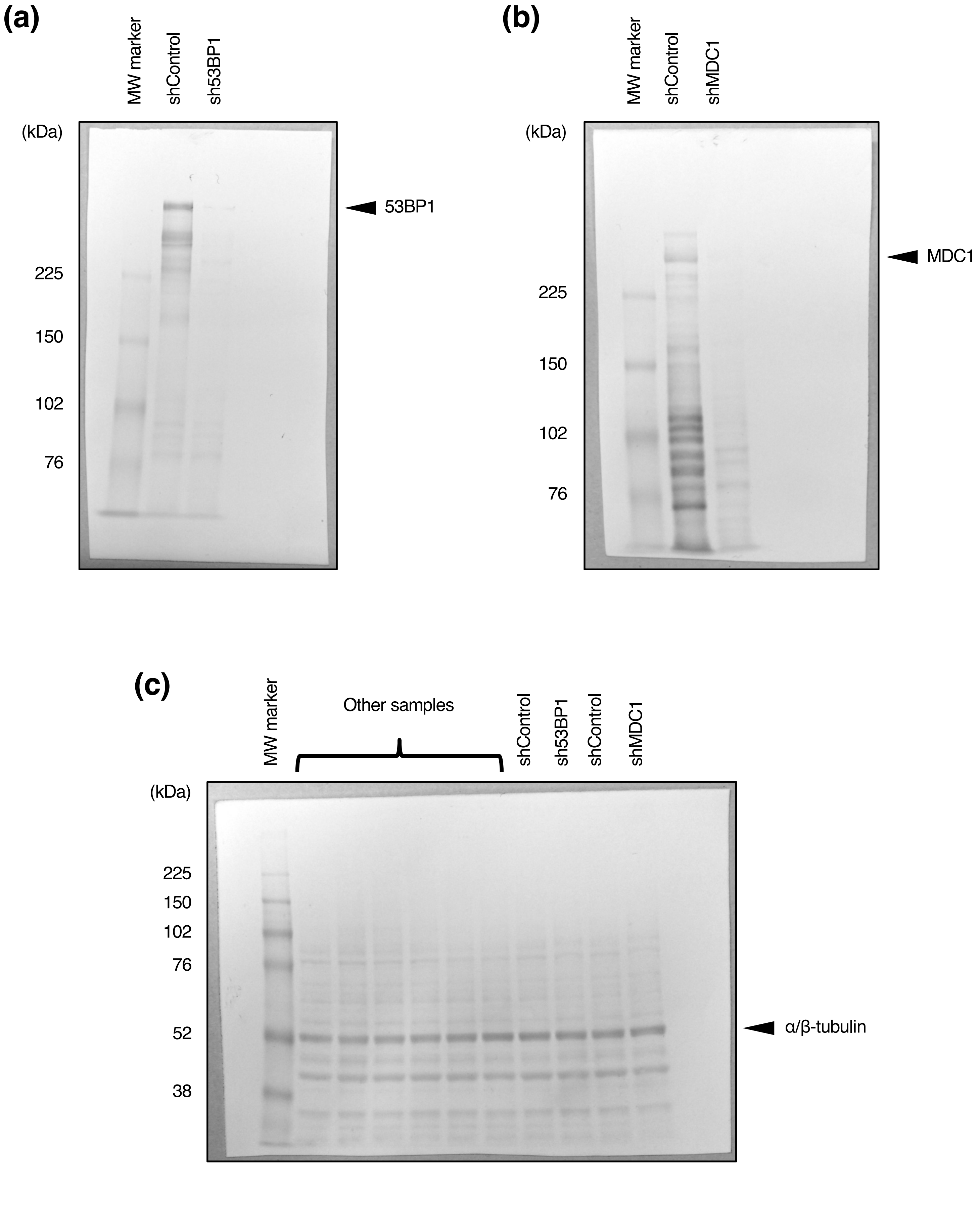

Supplementary Figure S9

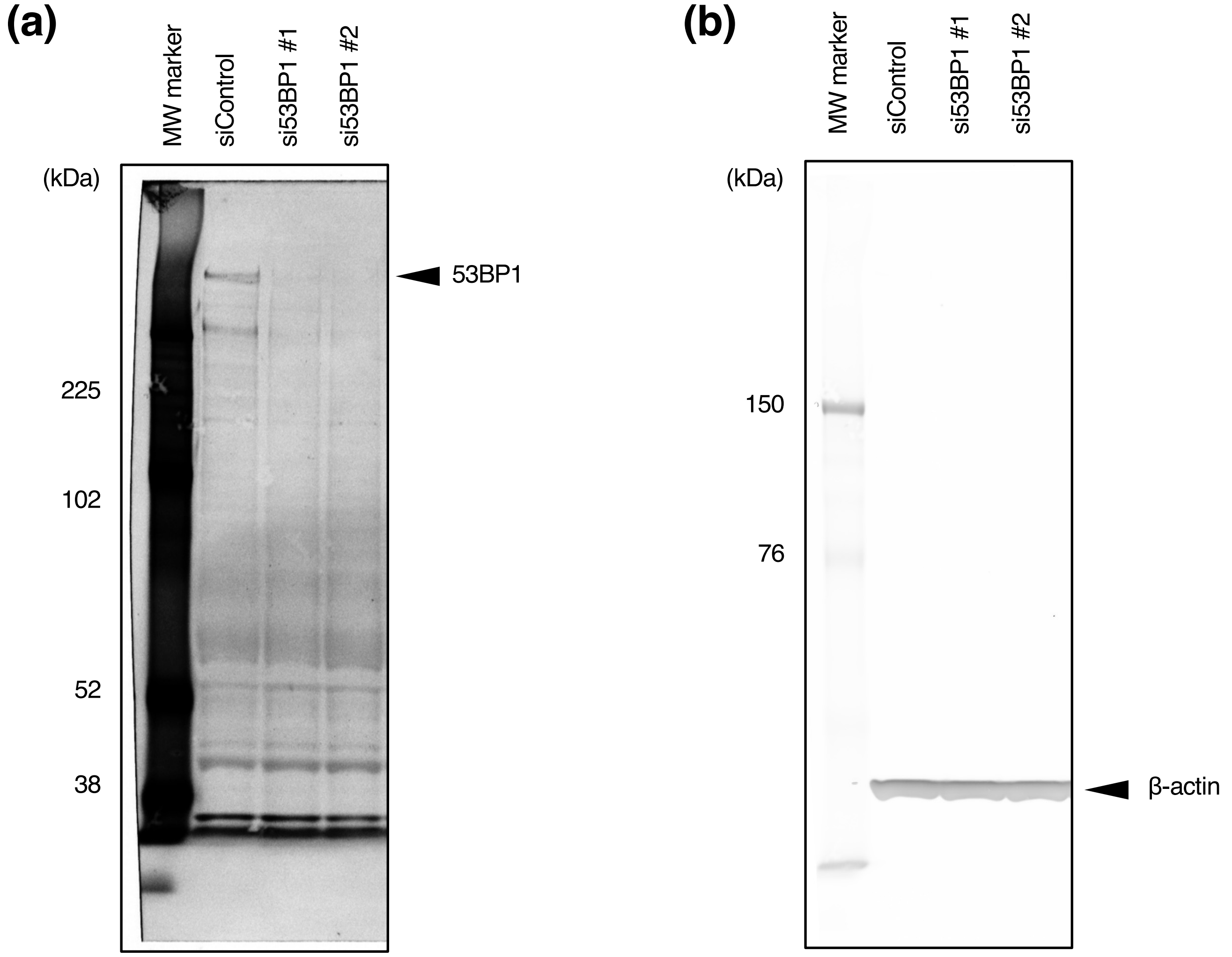

Supplementary Figure S10

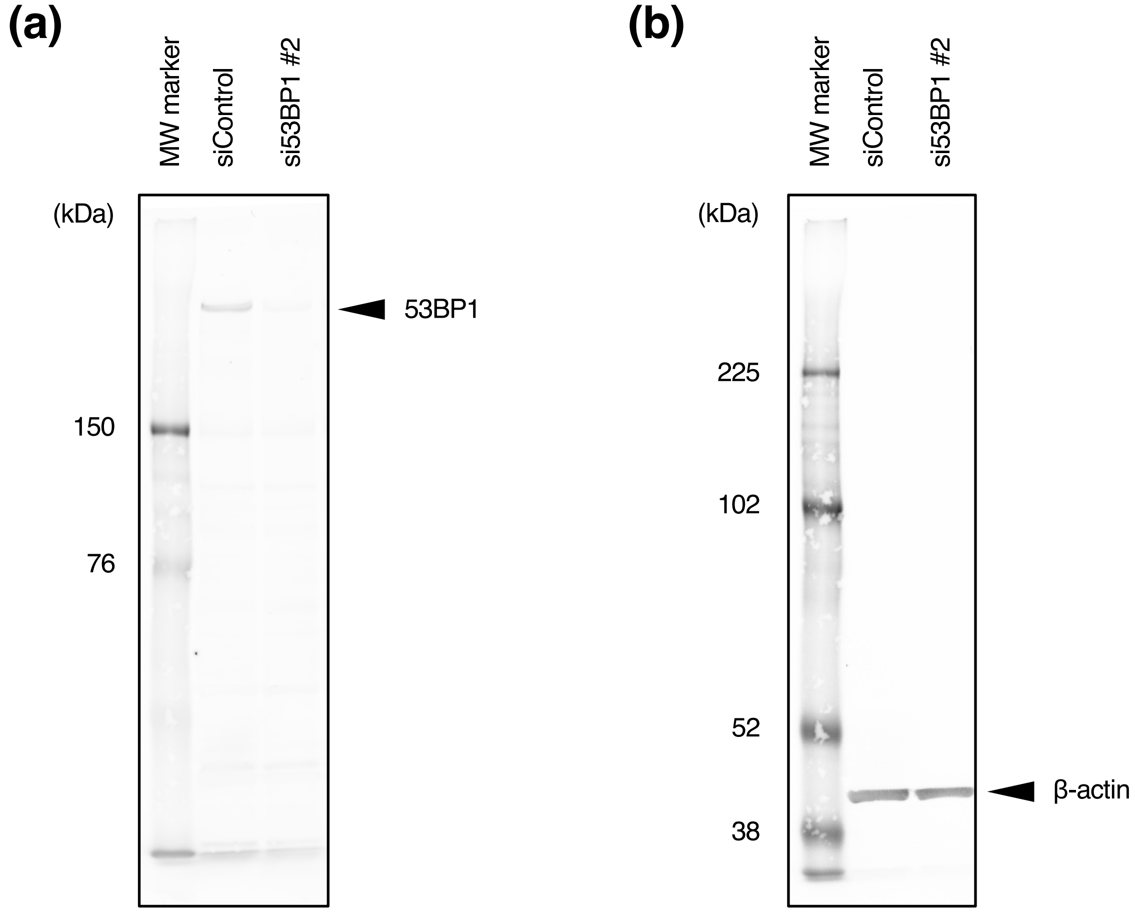

Supplementary Figure S11

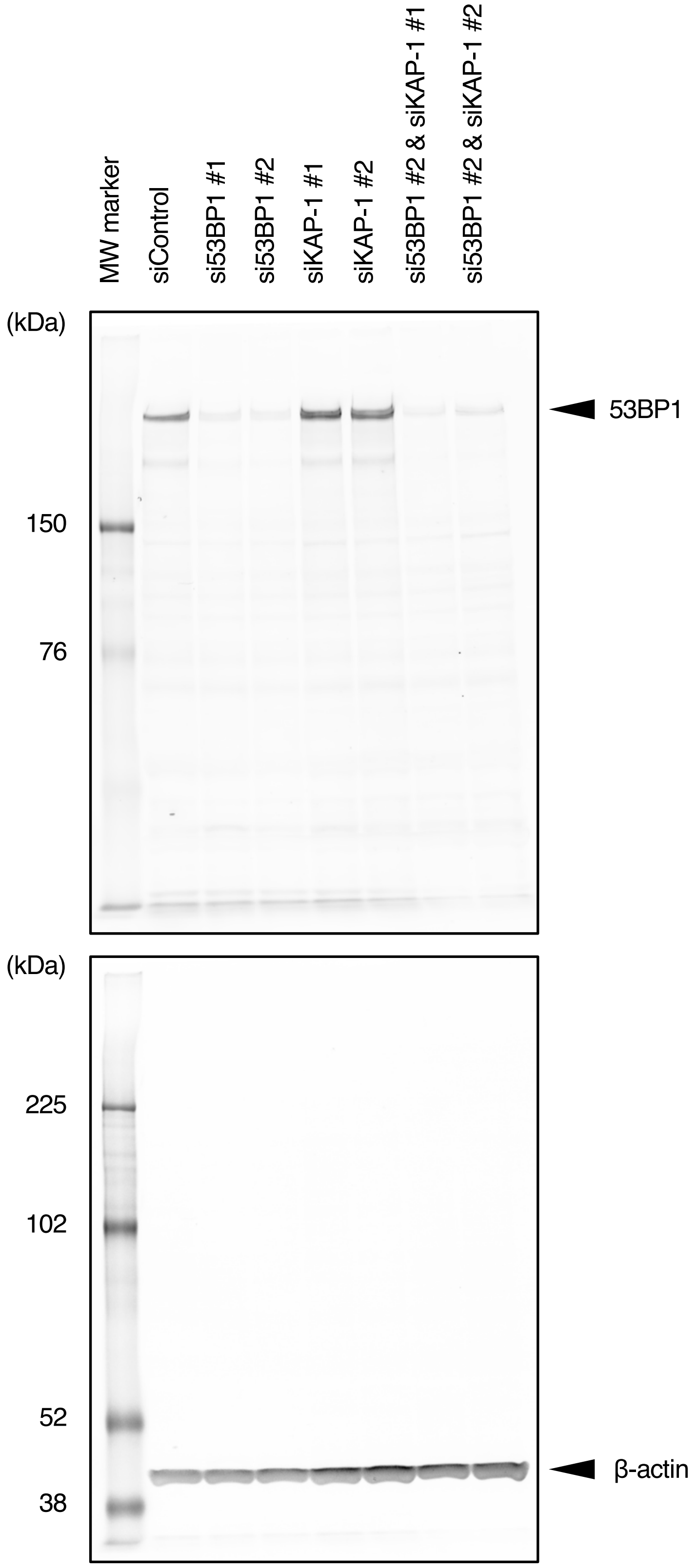

Supplementary Figure S12

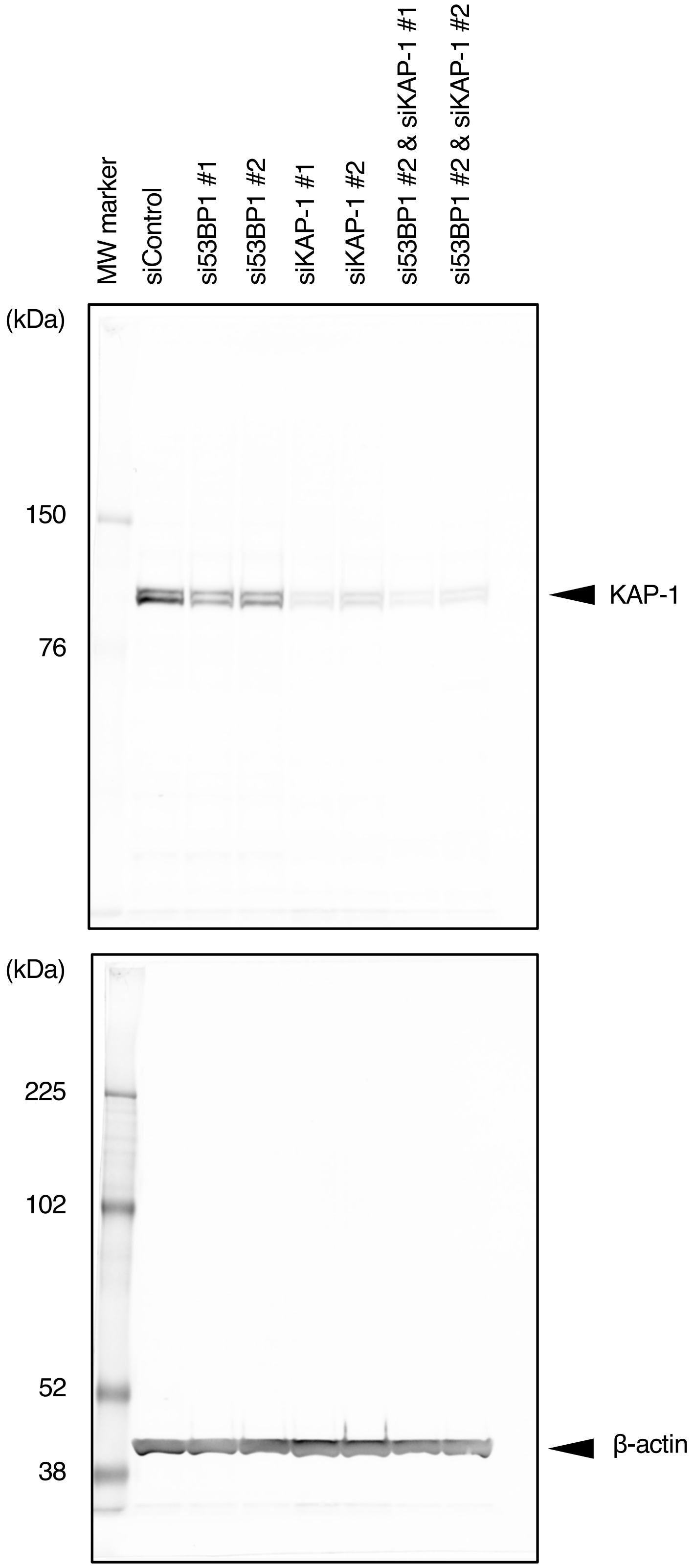

Supplementary Figure S13

Target genes	siRNA sequences
MRE11	siMRE11 (for human, ON-TARGETplus SMARTpool, GE Life Sciences, USA) is a mixture of the following four siRNAs:<br>5’-GGAGGUACGUCGUUUCAGA-3’<br>5’-GGAAAUGAUACGUUUGUAA-3’<br>5’-CGAAAUGUCACUACUAAGA-3’<br>5’-GAAAGGCUCUAUCGAAUGU-3’
CtIP	siCtIP (for human, ON-TARGETplus SMARTpool, GE Life Sciences, USA) is a mixture of the following four siRNAs:<br>5’-GGAGCUACCUCUAGUAUCA-3’<br>5’-GAGGUUAUAUUAAGGAAGA-3’<br>5’-GAACAGAAUAGGACUGAGU-3’<br>5’-GCACGUUGCCCAAAGAUUC-3’
53BP1	si53BP1#1 (for human): 5’-GCUAUAUCCUUGAAGAUUUUU-3’<br>si53BP1#2 (for human and mouse): 5’-GGACUCCAGUGUUGUCAUUUU-3’<br>si53BP1#3 (for mouse): 5’-UACUCUGGUCAAACAAGUCCUCCUG-3’
KAP-1	siKAP-1#1 (for human, ON-TARGETplus SMARTpool, GE Life Sciences, USA) is a mixture of the following four siRNAs:<br>5’-GAACGAGGCCUUCGGUGAC-3’<br>5’-AGACAGCACUGGCGUGGUG-3’<br>5’-GCGAUCUGGUUAUGUGCAA-3’<br>5’-GAAAUGUGAGCGUGUACUG-3’<br>siKAP-1#2 (for human, Stealth siRNA, Life Technologies, USA):<br>5’-GCAUGAACCCCUUGUGCUGUUUUGU-3’
CHD3	siCHD3#1 (for human, Stealth siRNA, Life Technologies, USA):<br>5’-GGGCCAUCAUUCGUGAGAAUGAAUU-3’<br>siCHD3#2 (for human, Stealth siRNA, Life Technologies, USA):<br>5’-AGGCACAGGUGAAGUUCCAUGUUCU-3’.

Supplementary Table S1

Cells	Dose & time after IR	No. of cells analyzed	No. of total foci	No. of paired foci	% of paired foci	% of paired foci (+) cells
BJ-hTERT	2 Gy, 0.5 hr	100	3018	380	12.6	83
BJ-hTERT	2 Gy, 2 hr	100	1591	215	13.5	71
BJ-hTERT	2 Gy, 4 hr	100	1043	159	15.2	56
BJ-hTERT	2 Gy, 8 hr	100	753	132	17.5	49

Supplementary Table S2

Genotype	Dose & time after IR	No. of cells analyzed	No. of total foci	No. of paired foci	% of paired foci	% of paired foci (+) cells
WT#1	1 Gy, 0.5 hr	100	2615	203	7.8	62
WT#1	1 Gy, 2 hr	100	902	142	15.7	51
WT#1	1 Gy, 4 hr	100	430	90	20.9	39
WT#1	1 Gy, 8 hr	100	239	47	19.7	21
WT#1	20 Gy, 4 hr	100	4651	354	7.6	81
WT#1	30 Gy, 8 hr	100	4079	278	6.8	74
$Ku80^{-/-}$	1 Gy, 0.5 hr	100	3774	621	16.5	99
$Ku80^{-/-}$	1 Gy, 2 hr	100	3364	805	23.9	97
$Ku80^{-/-}$	1 Gy, 4 hr	100	3277	993	30.3	97
$Ku80^{-/-}$	1 Gy, 8 hr	100	3026	943	31.2	99

Supplementary Table S3

Genotype	Dose & time after IR	No. of cells analyzed	No. of total foci	No. of paired foci	% of paired foci	% of paired foci (+) cells
WT#2	1 Gy, 0.5 hr	100	2281	279	12.2	75
WT#2	1 Gy, 2 hr	100	907	126	13.9	48
WT#2	1 Gy, 4 hr	100	483	100	20.7	42
WT#2	1 Gy, 8 hr	100	234	53	22.6	26
WT#2	15 Gy, 4 hr	100	2403	255	10.6	69
WT#2	20 Gy, 8 hr	100	1852	144	7.8	50
DNA-PKcs$^{-/-}$	1 Gy, 0.5 hr	100	3383	475	14.0	92
DNA-PKcs$^{-/-}$	1 Gy, 2 hr	100	2367	336	14.2	82
DNA-PKcs$^{-/-}$	1 Gy, 4 hr	100	1761	367	20.8	86
DNA-PKcs$^{-/-}$	1 Gy, 8 hr	100	1192	239	20.1	62

Supplementary Table S4

Cells	Dose & time after IR	No. of cells analyzed	No. of total foci	No. of paired foci	% of paired foci	% of paired foci (+) cells
2BN-hTERT	2 Gy, 0.5 hr	100	3878	211	5.4	66
2BN-hTERT	2 Gy, 2 hr	100	3802	247	6.5	69
2BN-hTERT	2 Gy, 4 hr	100	3474	270	7.8	76
2BN-hTERT	2 Gy, 8 hr	100	3205	314	9.8	76

Supplementary Table S5

siRNA	Dose & time after IR	No. of cells analyzed	No. of total foci	No. of paired foci	% of paired foci	% of paired foci (+) cells
siControl	2 Gy, 0.5 hr	100	3168	268	8.5	76
siControl	2 Gy, 2 hr	100	1653	143	8.7	50
siControl	2 Gy, 4 hr	100	983	94	9.6	38
siControl	2 Gy, 8 hr	100	477	68	14.3	30
siMRE11	2 Gy, 0.5 hr	100	2702	178	6.6	61
siMRE11	2 Gy, 2 hr	100	1624	155	9.5	56
siMRE11	2 Gy, 4 hr	100	933	86	9.2	35
siMRE11	2 Gy, 8 hr	100	528	67	12.7	30
siCtIP	2 Gy, 0.5 hr	100	3348	219	6.5	64
siCtIP	2 Gy, 2 hr	100	1874	149	8	50
siCtIP	2 Gy, 4 hr	100	1129	121	10.7	47
siCtIP	2 Gy, 8 hr	100	521	62	11.9	28

Supplementary Table S6

Cells	Dose & time after IR	No. of cells analyzed	No. of total foci	No. of paired foci	% of paired foci	% of paired foci (+) cells
AT5BI-hTERT	2 Gy, 0.5 hr	100	3454	520	15.1	91
AT5BI-hTERT	2 Gy, 2 hr	100	2158	385	17.8	87
AT5BI-hTERT	2 Gy, 4 hr	100	1592	330	20.7	79
AT5BI-hTERT	2 Gy, 8 hr	100	1372	304	22.2	75

Supplementary Table S7

siRNA	Dose & time after IR	No. of cells analyzed	No. of total foci	No. of paired foci	% of paired foci	% of paired foci (+) cells
siControl	2 Gy, 2 hr	100	2684	256	9.5	74
siControl	2 Gy, 4 hr	100	1712	246	14.4	74
siControl	2 Gy, 8 hr	100	1235	186	15.1	65
si53BP1 #1	2 Gy, 2 hr	100	2985	177	5.9	61
si53BP1 #1	2 Gy, 4 hr	100	1992	111	5.6	44
si53BP1 #1	2 Gy, 8 hr	100	1141	103	9	40
si53BP1 #2	2 Gy, 8 hr	100	1053	99	9.4	42

Supplementary Table S8

siRNA	Chromatin	Dose & time after IR	No. of cells analyzed	No. of total foci	No. of paired foci	% of paired foci	% of paired foci (+) cells
siControl	EC	6 Gy, 8 hr	92	582	121	20.8	44.6
siControl	HC	6 Gy, 8 hr	94	429	133	31.0	55.3
si53BP1 #2	EC	6 Gy, 8 hr	75	574	36	6.3	22.7
si53BP1 #2	HC	6 Gy, 8 hr	76	426	35	8.2	19.7

Supplementary Table S9

siRNA	Dose & time after IR	No. of cells analyzed	No. of total foci	No. of paired foci	% of paired foci	% of paired foci (+) cells
siControl	6 Gy, 8 hr	100	1687	209	12.4	70
si53BP1 #2	6 Gy, 8 hr	100	1978	104	5.3	43
siKAP-1 #1	6 Gy, 8 hr	100	2013	220	10.9	66
si53BP1 #2 + siKAP-1 #1	6 Gy, 8 hr	100	2535	245	9.7	77
siKAP-1 #2	6 Gy, 8 hr	100	1831	76	4.2	31
si53BP1 #2 + siKAP-1 #2	6 Gy, 8 hr	100	2092	187	8.9	63
siCHD3 #1	6 Gy, 8 hr	100	1756	129	7.3	48
si53BP1 #2 + siCHD3 #1	6 Gy, 8 hr	100	2417	234	9.7	62
siCHD3 #2	6 Gy, 8 hr	100	1839	148	8	54
si53BP1 #2 + siCHD3 #2	6 Gy, 8 hr	100	2359	241	10.2	72
